# Supplementary material for: Nellie: automated organelle segmentation, tracking and hierarchical feature extraction in 2D/3D live-cell microscopy
Source: Nat Methods. 2025 Feb 27;22(4):751–63. doi: 10.1038/s41592-025-02612-7 (PMC11978511; doi:10.1038/s41592-025-02612-7)
Supplement: Supplementary file 1 — Supplementary Notes 1–14, Figs. 1–8 and Table 1. [file 41592_2025_2612_MOESM1_ESM.pdf]

nature methods

Supplementary information https://doi.org/10.1038/s41592-025-02612-7

Nellie: automated organelle segmentation, tracking and hierarchical feature extraction in 2D/3D live-cell microscopy

In the format provided by the authors and unedited

Table of Contents

Supplementary Note 1: Runtime benchmarking of intracellular segmentation and tracking algorithms ... 3

Supplementary Note 2: Nellie's extended preprocessing pipeline ... 6

Supplementary Data Fig. 1: Contributions of preprocessing multi-scale intermediates ... 17

Supplementary Data Fig. 2: Frangi filter parameter effects on 3D structural contrast enhancement ... 18

Supplementary Note 3: The Minotri threshold ... 19

Supplementary Note 4: Segmentation comparison and benchmarking against state-of-the-art organelle segmentation algorithms ... 22

Supplementary Note 5: The Swin UNETR Deep Learning Model Architecture for Organelle Segmentation in Fluorescence Microscopy Images ... 30

Supplementary Data Fig. 3: Training and validation metrics for Swin UNETR segmentation models ... 40

Supplementary Data Fig. 4: Visualizations of segmentations from Nellie and custom trained Swin UNETR models ... 41

Supplementary Note 6: Nellie's extended segmentation pipeline ... 41

Supplementary Note 7: Nellie's mocap marker generation pipeline via multi-scale and adaptive local maxima detection ... 44

Supplementary Data Fig. 5: Computationally efficient distance transformation using segmentation border coordinate k-d trees ... 47

Supplementary Data Fig. 6: Multi-scale local maxima peak detection for mocap marking ... 48

Supplementary Note 8: Efficient distance transformation using k-dimensional trees ... 49

Supplementary Note 9: Tracking comparison and benchmarking against state-of-the-art organelle tracking algorithms ... 51

Supplementary Note 10: Creation of feature-based cost matrix for inter-frame mocap marker linkage ... 59

Supplementary Note 11: Temporal continuity in organelle tracking via forward and backward interpolation of semantic segmentations across frames ... 61

Supplementary Note 12: Spatial and temporal feature extraction of hierarchical organelle levels, extended ... 64

Supplementary Table 1: Table of Nellie's output features ... 69

Supplementary Data Fig. 7: Additional performance metrics from organelle unmixing random forest classifiers ... 72

Supplementary Note 13: Multi-mesh graph-based representation of cellular organelle networks.. 73

Supplementary Note 14: Graph autoencoder model construction for organelle multi-mesh network analysis........ 76

Supplementary Data Fig. 8: Loss curves for the Multi-mesh GNN........ 81

References........ 82

Supplementary Note 1: Runtime benchmarking of intracellular segmentation and tracking algorithms

Here we test segmentation and tracking runtimes of Nellie and other SOTA segmentation and tracking tools on datasets of increasing sizes (Extended Fig. 1a, b).

All segmentation pipelines were executed as described in Supplementary Note 4, with consistent methodologies applied across the various tools for a fair comparison. The runtime performance of each segmentation algorithm—MitoGraph, Mitometer, Nellie (CPU), and Nellie (GPU)—was tested on increasingly larger datasets, ranging from 3.7 MB to 1.87 GB, to evaluate their scalability and efficiency.

For Nellie, the segmentation process comprises multiple stages, including pre-processing, semantic segmentation, instance segmentation, skeletonization, and branch relabeling. The combined runtime of these stages is reported as the total segmentation time. To test the performance on larger datasets, we used two temporal frames of a dataset representing a single yeast cell with fluorescently labeled mitochondria and mirrored the dataset laterally to double its size with each subsequent run (Extended Fig. 1c, d). This methodology ensured a fair assessment of how each tool handles progressively larger 3D datasets, starting from the initial 3.7 MB and scaling up to 1.87 GB.

The results show that Nellie, running on either the CPU or GPU vastly outperforms both MitoGraph and Mitometer in terms of segmentation speed. Notably, Nellie exhibits a

near-linear increase in segmentation time as dataset size grows, demonstrating excellent scalability and computational efficiency. For the smallest dataset (3.7 MB), for both CPU and GPU runs, Nellie completes segmentation in under 5 seconds, while MitoGraph requires nearly 40 seconds, and Mitometer requires approximately 10 seconds. This difference becomes even more pronounced as the dataset size increases. At 233.1 MB, Nellie (GPU) finishes the segmentation task in roughly 1 minute and Nellie (CPU) finishes in just over 7 minutes, whereas MitoGraph takes close to 45 minutes and Mitometer requires close to 10 minutes. At the largest dataset size tested, 1.87 GB, Nellie (GPU) completes segmentation in 9 minutes and Nellie (CPU) in just under an hour, maintaining a steady performance across larger datasets. In stark contrast, MitoGraph requires over 17 hours to process the same dataset, and Mitometer takes just over an hour. These results highlight the large performance gains provided by Nellie when using GPU acceleration, particularly for large datasets, where the other tools struggle to maintain reasonable runtimes.

Similarly, the runtime performance of the different tools for tracking tasks follows a similar experimental setup. The tracking pipelines were executed as described in Supplementary Note 9, with MitoTNT relying on segmentation outputs from MitoGraph, due to the specific file format output from MitoGraph being required by MitoTNT, and Nellie and Mitometer using Nellie’s segmentation outputs for tracking mitochondria from frame 0 to frame 1 of the two temporal frames of the 3D dataset. For Nellie, tracking time reflects the combined runtime of the motion capture marking and matching pipelines.

Again, the results show that Nellie (GPU) vastly outperforms the other tools in terms of tracking time, particularly for larger datasets. For the smallest dataset (3.7 MB), Nellie completes tracking in less than 1 second over both the GPU and CPU, while MitoTNT requires nearly twice that time, and Mitometer about five times that time. As the dataset size increases to 233.1 MB, Nellie (GPU) completes the tracking task in about 11 seconds and Nellie (CPU) in roughly 1 minute, compared to nearly 20 minutes for MitoTNT and more than 5 minutes for Mitometer. For the largest dataset (1.87 GB), Nellie (GPU) completes the tracking task in less than 3 minutes and Nellie (CPU) in 14 minutes, whereas MitoTNT could not complete the tracking of the two largest datasets in less than a day (our cutoff), and Mitometer required just under 1 hour. These results further underscore the significant advantages of Nellie in terms of both segmentation and tracking efficiency, especially when deployed on GPU hardware.

These results demonstrate that Nellie, whether running on CPU or GPU, computationally outperforms MitoGraph and Mitometer in segmentation tasks, and MitoTNT and Mitometer in tracking tasks across a wide range of dataset sizes. The use of GPU acceleration provides an especially dramatic speedup, allowing Nellie to handle even the largest datasets tested in a fraction of the time required by the other tools. This makes Nellie a powerful and scalable tool for high-throughput organelle segmentation and tracking in 3D microscopy datasets, enabling researchers to process and analyze large volumes of data quickly and accurately.

Supplementary Note 2: Nellie's extended preprocessing pipeline

Optimization of multi-scale Gaussian filtering for anisotropic images in cellular fluorescence microscopy datasets

In our study, the establishment of minimum and maximum radii for filtering operations is guided by the specific parameters of the imaging metadata, ensuring an alignment with the physical dimensions represented within the dataset. The lower bound for the radius is determined by selecting the greater of two values: a predefined threshold of 0.20 micrometers, which approximates the diffraction limit of light, or the pixel size in the X dimension of the image. This approach ensures that the filtering scale remains relevant to the resolution characteristics of the dataset. Conversely, the upper bound is uniformly set at 1 micrometer, marking the maximal scale of interest for our analysis. These radii are subsequently translated into pixel/voxel dimensions. Mathematically, we can express this as:

$$r_{min} = max(0.2um,\ pixel\ size_X)$$

$$r_{max} = 1.00\ um$$

$$r_{min\ px} = \frac{r_{min}}{pixl\ size_X}$$

$$r_{max\ px} = \frac{r_{max}}{pixel\ size_X}$$

For the Gaussian filtering process, we derive the minimum and maximum sigma values directly from these pixel-based radii, applying reduction factors of one-half and one-third, respectively. This strategy is designed to calibrate the filter's sensitivity to features of varying intracellular sizes:

$$\sigma_{min} = min\left(\frac{r_{min\,px}}{2}, \frac{r_{max\,px}}{3}\right)$$

$$\sigma_{max} = max\left(\frac{r_{min\,px}}{2}, \frac{r_{max\,px}}{3}\right)$$

To span the range of feature scales, we implement a series of five sigma values, ensuring comprehensive coverage across the standard deviation spectrum. The inter-sigma step size is calculated to bridge the desired feature scales effectively, imposing a minimum threshold on step size to prevent redundantly small increments that yield negligible changes in filtering outcomes:

$$N_{\sigma} = 5$$

$$step\ size_{\sigma} = max\left(0.2, \frac{\sigma_{max} - \sigma_{min}}{N_{\sigma}}\right)$$

$$\sigma_{array} = [\sigma_{min}, \sigma_{min} + step\ size_{\sigma}, \ldots, \sigma_{max}]$$

This structured approach yields a discrete set of sigma values, each corresponding to a specific standard deviation within the Gaussian filtering framework, thus enabling the filter to adaptively respond to biological features of different scales.

In addressing the challenge of anisotropic datasets prevalent in microscopy imaging – characterized by equivalent resolutions in the X and Y axes but reduced resolution along the Z axis – it is imperative to adjust the kernel values of 3D convolutional filters. This adjustment is critical within our preprocessing framework, which utilizes Gaussian filters across a multi-scale parameter space in both Frangi and Laplacian of Gaussian filtering applications. To counteract anisotropy, we construct a sigma vector for each scale, maintaining uniform standard deviations for the X and Y dimensions while

adapting the Z dimension's standard deviation in accordance with the voxel resolution ratio (Z/X). This methodology ensures a consistent filtering effect across the dataset, irrespective of inherent resolution variances. Mathematically, for a given sigma value σ, we define the sigma vector as:

$$\sigma_{vec3} = \left(\sigma \frac{res_X}{res_Z}, \sigma, \sigma\right)$$

In 2D datasets, which are typically (and for the use of Nellie, we assume to be) exempt from anisotropic concerns, a singular sigma value is employed without modifications for anisotropy:

$$\sigma_{vec2} = (\sigma, \sigma)$$

Automation of the Frangi filter for optimized contrast enhancement of intracellular structures

For each sigma value of our Gaussian filter, several steps are undertaken to both automate and optimize the Frangi filter to enhance the contrast of structures at the intracellular scale. First, the gamma parameter for the Frangi filter is derived from the minimum of the triangle and Otsu threshold values – termed henceforth as the Minotri threshold – of the Gaussian filtered image, providing a size-adaptive approach to contrast enhancement of structural features (Supplementary Note 3)[1,2]:

$$\gamma_\sigma = min\left(T_{tri}\left(I_\sigma\right), T_{Otsu}\left(I_\sigma\right)\right)$$

where I_σ is the Gaussian filtered image at scale σ, and T_{tri} and T_{Otsu} are the triangle and Otsu thresholding functions, respectively.

Next, the Hessian matrix for each sigma level is computed:

$$H_{\sigma} = [$$

$$\left[\frac{\delta^2 I_{\sigma}}{\delta x^2}, \frac{\delta^2 I_{\sigma}}{\delta y \delta x}, \frac{\delta^2 I_{\sigma}}{\delta y \delta z}\right]$$

$$\left[\frac{\delta^2 I_{\sigma}}{\delta y \delta x}, \frac{\delta^2 I_{\sigma}}{\delta y^2}, \frac{\delta^2 I_{\sigma}}{\delta y \delta z}\right]$$

$$\left[\frac{\delta^2 I_{\sigma}}{\delta z \delta x}, \frac{\delta^2 I_{\sigma}}{\delta z \delta y}, \frac{\delta^2 I_{\sigma}}{\delta z^2}\right]$$

$$]$$

The Hessian matrix is rescaled and its Frobenius norm is calculated:

$$H_{\sigma\, rescaled} = \frac{H_{\sigma}}{max\left(\left|H_{\sigma}\right|\right)}$$

$$F_{\sigma} = \left|\left|H_{\sigma\, rescaled}\right|\right|$$

The Hessian matrix is masked using the Minotri threshold of the Frobenius norm:

$$Mask_{\sigma} = F_{\sigma} > min\left(T_{tri}\left(F_{\sigma}\right), T_{Otsu}\left(F_{\sigma}\right)\right)$$

$$H_{\sigma\, masked} = H_{\sigma} \cdot Mask_{\sigma}$$

which is a modified approach to MitoGraph's masking method[3]. This approach not only significantly improves computational and memory efficiency, which is particularly crucial for eigenvalue calculations of large datasets, but also allows for completely automatic parameter selection. The eigenvalues of the vectorized Hessian matrix are calculated in memory-efficient chunks and sorted by their absolute values within individual voxels:

$$\lambda_{1,\sigma}, \lambda_{2,\sigma}, \lambda_{3,\sigma} = sort\left(\left|eigvals\left(H_{\sigma\, masked}\right)\right|\right)$$

where $\left|\lambda_{1,\sigma}\right| \leq \left|\lambda_{2,\sigma}\right| \leq \left|\lambda_{3,\sigma}\right|$

Finally, Frangi dissimilarity metrics are calculated:

$$R_{A,\sigma} = \frac{\left|\lambda_{2,\sigma}\right|}{\left|\lambda_{3,\sigma}\right|}$$

$$R_{B,\sigma} = \frac{\left|\lambda_{1,\sigma}\right|}{\sqrt{\left|\lambda_{2,\sigma} \cdot \lambda_{3,\sigma}\right|}}$$

$$S_{\sigma} = \sqrt{\lambda_{1,\sigma}^{2} + \lambda_{2,\sigma}^{2} + \lambda_{3,\sigma}^{2}}$$

The vesselness measure V_{σ} at scale σ is computed:

$$V_{\sigma} = \left(1 - e^{\frac{-R_{A,\sigma}^{2}}{2\alpha^{2}}}\right) \cdot \left(e^{\frac{-R_{B,\sigma}^{2}}{2\beta^{2}}}\right) \cdot \left(1 - e^{\frac{-S_{\sigma}^{2}}{2\gamma_{\sigma}^{2}}}\right)$$

where α and β are shape-balanced parameters described in detail in the next section, and γ is the scale-adaptive parameter derived above. These metrics as a whole are used to calculate the inherent structural measure of the organelles at that specific scale, rather than the traditional approach of using the same metrics across all scales.

The final multi-scale structure-enhanced filtered image – termed henceforth as the preprocessed image – is constructed by compiling the maximum voxel-wise value across all scales, ensuring that the most prominent features at each scale are captured in the final image (Fig. 1e, Supplementary Data Fig. 1):

$$I_{pre} = max_{\sigma}\left(V_{\sigma}\right)$$

Automated selection of the Frangi filter parameters

Though the Frangi filter, which uses the eigenvalues of the Hessian matrix of the image as attributes, is historically used to accentuate vessel structures within images, it also

works exceptionally well at accentuating structural components in general, as long as proper Frangi parameters and scale ranges over which to run the filter are chosen. As organelle shapes vary dramatically both between and within different types of organelles, we must carefully balance the Frangi filter's parameters and scale range in order to properly capture this diversity without accentuating any specific type of organelle. In this regard, we explicitly and specifically modify the Frangi filter to allow for automatic adaptation to specific image contexts, including and beyond scale variations.

The alpha parameter controls the sensitivity of the filter to the deviation from a blob-like structure. In the context of structure detection, it helps in distinguishing between disc-like, blob-like, and tube-like structures based on the eigenvalues of the Hessian. In practice, a lower alpha value makes the filter more sensitive to disc-like structures and less sensitive to tube-like structures, while increasing alpha will do the opposite, making the filter less responsive to blob-like and disc-like structures and more focused on tube-like features. We set:

$$\alpha^2 = 0.5$$

This setting provides a moderate sensitivity to blob-like structures (Supplementary Data Fig. 2). This allows the filter to be balanced in its response to tube-like structures and blob-like structures. This setting means the filter will enhance tubular structures while still allowing for some degree of response to blob-like features. It won't be overly discriminative against structures that slightly deviate from the traditionally Frangi-directed tubular shape.

The beta parameter controls the filter's sensitivity to the deviation from a disc-like structure. It is directly involved in the suppression of the response of the filter to disc-like and blob-like structures. In practice, adjusting beta alters the filter's response to different types of structures. A lower beta value increases the sensitivity of the filter to disc-like structures, while a higher beta value makes the filter more selective for tubular structures. We also set:

$$\beta^2 = 0.5$$

This setting provides a moderate level of suppression for disc-like structures (Supplementary Data Fig. 2). This prevents the filter from being overly aggressive in suppressing disc-like features.

We modify the calculation of R_B, which measures the deviation from a line-like structure:

$$R_{B,\sigma} = \frac{|\lambda_{2,\sigma}|}{\sqrt{|\lambda_{2,\sigma} \cdot \lambda_{3,\sigma}|}}$$

where $\lambda_{1,\sigma}, \lambda_{2,\sigma}, \lambda_{3,\sigma}$ are the eigenvalues of the Hessian matrix, sorted such that $|\lambda_{1,\sigma}| \leq |\lambda_{2,\sigma}| \leq |\lambda_{3,\sigma}|$.

This results in the filter deemphasizing purely line-like and tube-like structures and emphasizing both line and blob-like structures. Running this change through our segmentation validation comparison studies showed slightly improved F1 and IoU scores on the multi grid test, and no changes in the pixel size and separation tests.

The gamma parameter is a scaling parameter that controls the sensitivity of the filter to the overall magnitude of the eigenvalues. This parameter essentially acts as a normalization factor in the function. In practice, changing gamma affects the filter's sensitivity to the background noise and contrast of the image. A higher gamma value can suppress the response of the filter to areas with low contrast, reducing the likelihood of detecting false positives due to noise. Conversely, a lower gamma would increase sensitivity to lower contrast regions but would also increase vulnerability to noise. This is the most important parameter to set properly, as poor gamma selection will lead to failure in correctly thresholding regions of interest post-filtration. By automating the gamma selection process by deriving it from the Minotri threshold, we're linking gamma to the inherent contrast characteristics of the image at that scale. This is a novel way to make the filter's response adaptive to each specific scale's contrast profile. This is particularly useful where samples can vary greatly in their staining, illumination, and intrinsic structural contrast. Additionally, by adapting gamma using our Minotri threshold method at each scale independently, we automatically adjust gamma to enhance subtler structures in low-contrast images, while in high-contrast images, it prevents over-enhancement and preserves detail. This ensures that the filter's sensitivity to the eigenvalue magnitudes is tailored not just to the image's overall contrast profile, but also to the contrast profile relevant to structures at each specific scale. This means for finer scales (smaller Gaussian sigma) which targets smaller structures, the gamma value will adapt to the contrast nuances at that scale, and similarly for larger scales.

Preprocessing refinement techniques for structural contrast enhancement

Following the initial multi-scale structural enhancement of organelles, the pipeline incorporates further post-preprocessing techniques to refine the output images. The refined post-preprocessing begins with the application of a masking technique to the preprocessed image, aiming to eliminate regions with minimal structural information. This is achieved by setting a threshold that masks regions falling below the 1st percentile of non-zero pixel values:

$$T = percentile(I_{pre}, 1)$$

where percentile(A, p) returns the pth percentile of the non-zero values in A. From this threshold, the initial mask is created:

$$M(x, y, z) = \left\{1\ if\ I_{pre}(x, y, z) > T\ else\ 0\right\}$$

For 2D images, this simplifies to:

$$M(x, y) = \left\{1\ if\ I_{pre}(x, y) > T\ else\ 0\right\}$$

This ensures a focus on regions rich in meaningful content. Subsequently, a binary opening operation is performed on the mask to smooth out noise within the structures. This morphological operation, involving erosion followed by dilation, refines the mask's boundaries, and removes spurious pixels that could detract from the integrity of the analysis:

Let S be the structuring element. For 3D images, S is a 2x2x2 cube, while for 2D images, S is a 2x2 square.

For erosion:

$$E = M \ominus S = \left\{z | S_z \subseteq M\right\}$$

where S_z is the translation of S by vector z.

For dilation:

$$D = E \oplus S = \left\{ z \mid \widehat{S}_z \cap E \neq \varnothing \right\}$$

where $\widehat{S}_z$ is the reflection of S.

The final refined mask is the result of the binary opening:

$$M_{refined} = (M \ominus S) \oplus S$$

The final refined preprocessed image I_refined is obtained by applying the refined mask to the original preprocessed image:

$$I_{refined}(x, y, z) = I_{pre}(x, y, z) \cdot M_{refined}(x, y, z)$$

For 2D images:

$$I_{refined}(x, y) = I_{pre}(x, y) \cdot M_{refined}(x, y)$$

These post-preprocessing steps collectively ensure that the pipeline produces refined output images. This approach not only enhances the visibility of organelles but also prepares the images for accurate segmentations of regions of genuine structural significance.

2D datasets typically have reduced structural noise compared to 3D datasets, which can be attributed to several potential factors, including the reduced complexity of organelles that can be captured in a single plane, compared to the three-dimensional complexity of organelles in ZYX datasets. Furthermore, anisotropy present in 3D datasets - where differences in resolution between the XY plane and the Z-axis lead to

variability in structural clarity - typically necessitates more complex filtering approaches to achieve uniform enhancement across all dimensions.

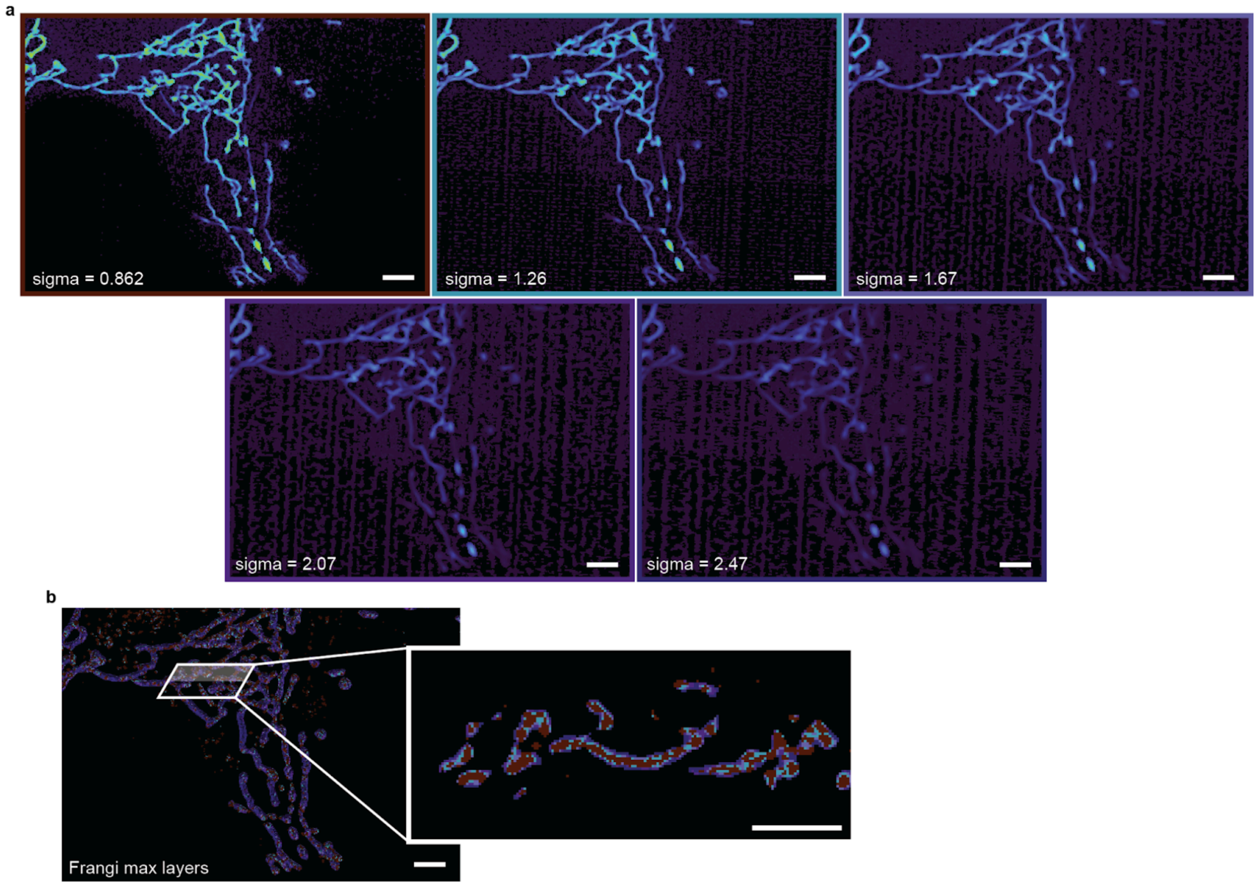

Supplementary Data Fig. 1: Contributions of preprocessing multi-scale intermediates
a, A 3D fluorescently mitochondrial-labeled cell run through a Frangi filter at 5 different scales, undergoing Gaussian filtration with respective sigma values. **b**, An integer-label image where the voxel color represents the corresponding Frangi filter scale contributor, as denoted by the border color. Scale bars are 5 um in length.

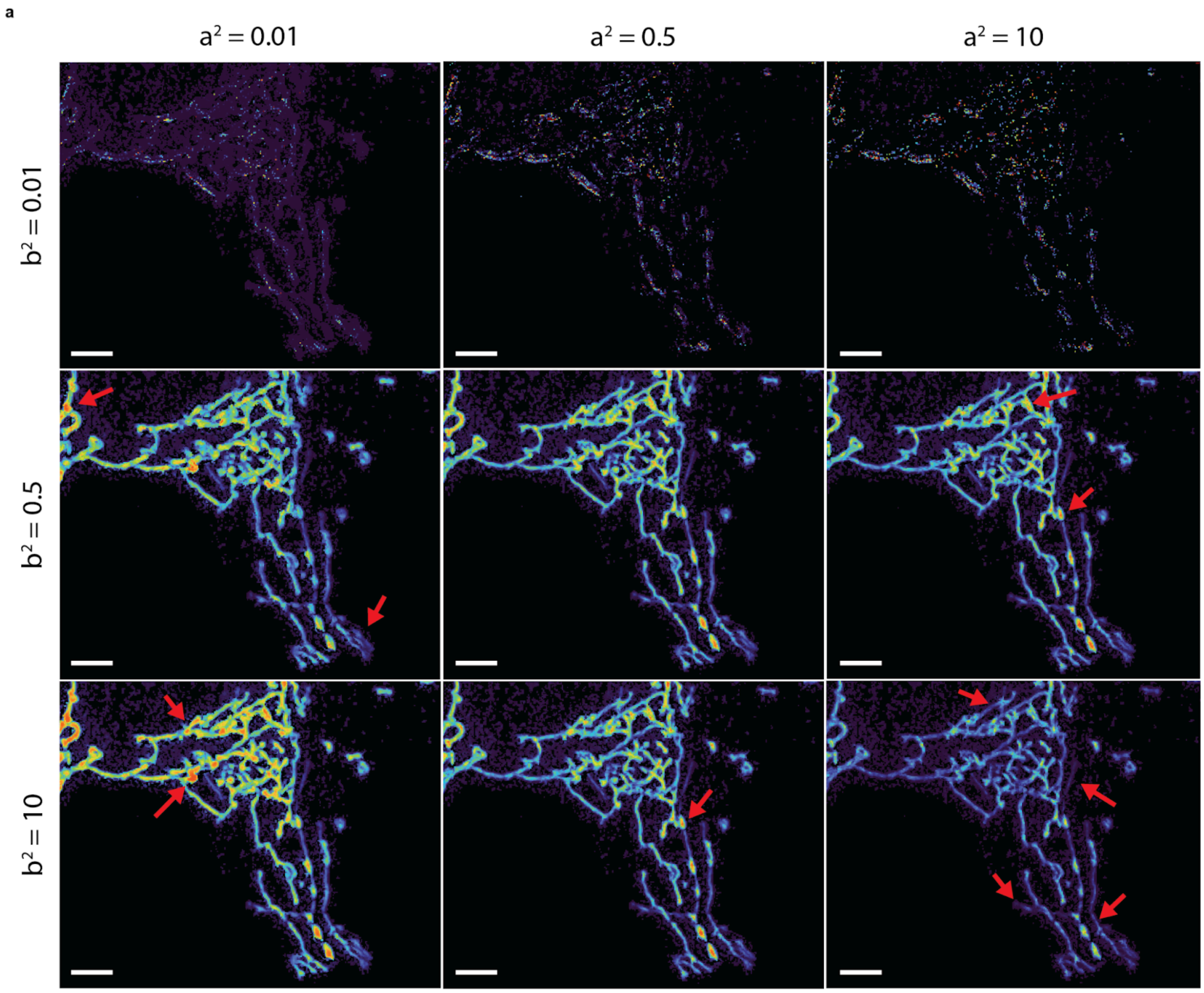

Supplementary Data Fig. 2: Frangi filter parameter effects on 3D structural contrast enhancement
a, A 3D fluorescently mitochondrial-labeled cell showcasing regions of tubular and blob-like structures. The outputs of the structural enhancement preprocessing using alpha and beta-squared Frangi filter parameters with alpha-squared ranging from 0.01 to 10 (columns), and beta-squared ranging from 0.01 to 10 (rows). The middle panel (0.5, 0.5) represents the hard-coded parameter set used in Nellie's pipeline. Red filled-in arrows show problematic regions. Scale bars are 5 um in length.

Supplementary Note 3: The Minotri threshold

In this study, we introduce the Minotri threshold approach - a fusion of the Otsu and Triangle methods, adapted to the unique challenges of automated organelle segmentation. The Minotri threshold is defined as:

$$T_{minotri} = min\left(T_{tri}, T_{Otsu}\right)$$

where T_{tri} is the Triangle threshold and T_{Otsu} is the Otsu threshold.

The Otsu method is well known for its capability to segment images based on a bimodal intensity distribution. It accomplishes this by calculating a threshold that maximizes the between-class variance, effectively distinguishing between the foreground (organelles) and the background:

Let the image have L gray levels [1, 2, ..., L]. The number of pixels at level i is denoted by n_i and the total number of pixels by N = n_1 + n_2 + ... + n_L.

Compute histogram and probabilities of each intensity level:

$$p_i = \frac{n_i}{N}$$

Compute cumulative sums for k = 0, 1, 2, ..., L-1:

$$P_1(k) = \sum_{i=1}^{k} p_i$$

Compute cumulative means:

$$m(k) = \sum_{i=1}^{k} \frac{i \cdot p_i}{P_i(k)}$$

Compute global intensity mean:

$$m_G = \sum_{i=1}^{L} i \cdot p_i$$

Compute between-class variance:

$$\sigma_B^2(k) = \frac{\left[m_G P_1(k) - m(k)\right]^2}{P_1(k)\left[1 - P_1(k)\right]}$$

The Otsu threshold is the value that maximizes between-class variance:

$$k^{\star} = argmax_k\left(\sigma_B^2(k)\right)$$

Otsu's algorithm excels in scenarios where the histogram exhibits two distinct peaks, corresponding to these two classes. In such cases, it tends to favor the class with the larger peak, thus ensuring the majority of the pixels from this peak are classified correctly. However, its performance may be less optimal in images with skewed histograms or those lacking a clear bimodal distribution.

On the other hand, the Triangle method approaches thresholding from a geometric perspective. It identifies the threshold by drawing a line from the peak of the histogram to the farthest end and then finding the point on the histogram that is furthest from this line:

Compute the histogram of the image.

Find the peak b_{max} of the histogram h.

Find the leftmost b_{min} and rightmost b_{max} non-zero bins of the histogram.

Compute the line equation passing through $\left(b_{min}, h\left[b_{min}\right]\right)$ and $\left(b_{max}, h\left[b_{max}\right]\right)$:

$$y = mx + c$$

where

$$m = \frac{h[b_{max}] - h[b_{min}]}{b_{max} - b_{min}}$$

$$c = h[b_{min}] - m \cdot b_{min}$$

For each bin i, compute the distance d_i from $(i, h[i])$ to the line:

$$d_i = \frac{|h[i]| - (mi + c)}{\sqrt{m^2 + 1}}$$

The Triangle threshold is the bin i that maximizes d_i:

$$i^{\star} = argmax_i(d_i)$$

This method is particularly effective in dealing with histograms that are not bimodal or are skewed, such as in cases where there is a smooth transition from foreground intensity to background intensity, or when there is a large amount of diffuse background signal, whose intensity lies within a distribution higher than the background but lower than the foreground, making it a versatile tool for images where Otsu's method might struggle.

Our Minotri threshold approach combines these two methodologies by adopting the minimum (min) of the thresholds determined by the Otsu (ot) and Triangle (tri) methods. This approach ensures a more nuanced and adaptable segmentation, particularly useful in microscopy images where intensity distributions can vary significantly. By choosing the lower threshold, we err on the side of inclusivity, capturing a broader range of (both photon density-based and structurally based) intensities that represent the organelles, while still maintaining a rigorous standard for segmentation quality. This strategy is

especially beneficial in cases where one method may yield a threshold that is too high, potentially excluding relevant structural information.

By integrating the Minotri threshold into our segmentation workflow, we achieve a balance between sensitivity and precision. This approach allows for the robust detection of structures across a wide range of intensity distributions, thereby enhancing the accuracy and reliability of our semantic segmentation process. This combined thresholding technique offers a versatile and effective tool for the detailed study of diverse organelles.

Supplementary Note 4: Segmentation comparison and benchmarking against state-of-the-art organelle segmentation algorithms

	Nellie (ours)	Mitometer	MitoGraph v3
**2D**	Yes	Yes	Yes
**3D**	Yes	Yes	Yes
**Temporal frames**	Optional	Required	Incompatible
**Metadata detection**	Yes	No	No
**Automated parameters**	Yes	Yes	No
**Node morphometrics**	Yes	No	Limited
**Branch morphometrics**	Yes	No	Limited
**Organelle morphometrics**	Yes	Yes	Limited
**Image-wide morphometrics**	Yes	No	No
**Mask output**	Yes	Yes	Limited
**Intermediates output**	Yes	Yes	Limited
**GUI**	Yes	Yes	No
**GPU-accelerated**	Yes	No	No
**Language**	Python	MATLAB	C++
**Compatible OS**	Mac, Windows, Linux	Mac, Windows, Linux	Mac

Nellie, 2024 (https://github.com/aelefebv/nellie)

The subject of this paper, Nellie is an automated segmentation, tracking, and hierarchical feature extraction pipeline for organelles in both 2D and 3D live-cell microscopy. Nellie optionally takes a time series, though the segmentation does not require it. Nellie automatically detects the metadata of the image if it is present, but allows the user to modify the metadata if needed. Nellie returns morphometrics at the node, branch, organelle, and image-wide level in .csv format. Nellie also returns all intermediates, including the preprocessed frangi-filtered image, the skeleton mask, the tip, junction, and edge image, the branch-labeled skeleton image, the branch-labeled segmentation image, and the organelle-labeled segmentation image. Nellie has a Napari-based GUI, and uses GPU acceleration for segmentation. Nellie is fully written in Python, and is compatible with all major operating systems.

Mitometer, 2021 (https://github.com/aelefebv/Mitometer)

Mitometer is an automated segmentation and tracking pipeline for mitochondria in both 2D and 3D live-cell microscopy, though still performs well for processing and analysis of other organelles. Mitometer requires temporal frames to perform segmentation, as it uses an inter-frame variability metric to determine smoothing and thresholding parameters. It requires the user to input image metadata such as the pixel resolution and the frame rate, and adjusts its segmentation pipeline accordingly. Mitometer's segmentation outputs are limited to connected-component analysis, but returns a wide variety of metrics in a .txt file. Mitometer also returns a time-series of the preprocessed diffuse background-removed image, and instance segmentations of individual

mitochondria, as well as a masked version of the original fluorescence intensity image. Mitometer has a GUI for both single-file and batch-file processing, but does not use GPU acceleration. It uses MATLAB, which is not open source, but is compatible with all major operating systems. For the sake of reproducibility in running benchmarking and comparisons, we have rewritten Mitometer's MATLAB segmentation algorithm in Python, with GPU acceleration. The rewritten code is accessible in the supplemental Nellie repository (https://github.com/aelefebv/nellie-supplemental).

MitoGraph v3, 2018 (https://github.com/vianamp/MitoGraph)
MitoGraph is an automated segmentation and analysis tool for 2D (since v3) and 3D mitochondrial images, though it works well for other organelles as well. Though the link to the code on the original webpage (https://rafelski.com/susanne/MitoGraph) no longer works, we assume that the first author's GitHub repository is a valid replacement. MitoGraph accepts, but does not segment images with multiple temporal frames. It also does not detect metadata automatically, and does not adjust the segmentation to the user-defined metadata (though it does scale the outputs accordingly). MitoGraph outputs a limited number of node, branch, and organelle morphometrics across various .mitograph, .cc, .txt, .gnet, and .coo formats. MitoGraph also outputs a 2D max projection of the post-segmentation image in .png format, as well as node, skeleton, and surface image intermediates in .vtk format. MitoGraph is written in C++, runs via the command line, and does not use GPU acceleration for segmentation. It is only compatible with MacOS. For the sake of reproducibility in running benchmarking and comparisons, we have written a batch conversion and processing script, adapted from

MitoTNT's scripts, as well as a method to reconstruct organellar objects by producing a sphere at each node position with its corresponding thickness, as we were unable to extract usable object masks for IoU and F1 segmentation quantification calculation without it. The scripts and methods are accessible in the supplemental Nellie repository (https://github.com/aelefebv/nellie-supplemental).

For the benchmarking and comparisons of the aforementioned segmentation algorithms, we chose to compare and contrast F1 scores to quantify accuracy in the detection of the correct objects, and the intersection over the union (IoU) to quantify the overlap of the algorithms' segmentation mask outputs and the ground truth object masks. As generating ground truth masks of 3D microscopy datasets is non-trivial and subject to user bias, we instead opt to build a suite of simulation tools to generate ground truth organelle-like objects. All of the simulation tools are made available in the supplemental Nellie repository (https://github.com/aelefebv/nellie-supplemental).

The first segmentation comparison tests each algorithm's ability to properly detect and segment objects of varying length, width, and intensities within the same image, considering organelles, even of the same type, may vary in size, length, and be tagged with intensity-varying functional readout markers. (Extended Data Fig. 2). To do this, we simulate a 3x510x510x510 (TZYX) OME-TIFF file. The dataset consists of 3 independently noise-sampled replicates of 10x10x10 simulated objects, which varies in thickness along Z, length along Y, and intensity along X. To simulate the object, we extend a single pixel line across Z, while simulating curvature in the Y and X axes. We

then perform a binary dilation, followed by a skeletonization to ensure a proper skeleton object is formed. Finally, we create a sphere with a diameter of the corresponding thickness at each skeleton pixel coordinate to create the mask of the object, and populate that mask with the corresponding intensity. Each object is contained within a 50x50x50 voxel cube, all of which get stitched together into the final test dataset. We generate these datasets with increasing background noise values, generated via both additive Gaussian and Poisonian distributions (Extended Data Fig. 2c,d). We hard code a resolution of 0.1 um/pixel. We use as input the full 3x510x510x510 dataset consisting of 1000 objects. We use Nellie's "skel_relabelled" image, MitoGraph's reconstructed image, and Mitometer's post-size exclusion mask as outputs. We additionally include both an Otsu and Triangle thresholded output for baseline. To calculate the F1 and IoU scores, we separate the output and ground truth datasets into the corresponding single-object 50x50x50 voxel cubes. For each single-object cube, we compare each connected component to the ground truth object and keep as the final IoU the maximum IoU found for each individual object. If the IoU is above the minimum IoU threshold (0.1) we calculate F1 using the number of connected components in the output image. We then aggregate the scores for all 1000 objects to calculate our final scores. We find that Nellie outperforms all other methods in IoU score across all noise levels, and is similar to Mitometer's F1 score at low noise levels, but outperforms at mid to high noise levels (Extended Data Fig. 2a,b).

The second segmentation comparison tests each algorithm's ability to segment and detect objects across varying voxel resolutions, considering microscopy datasets are

often acquired with widely varying parameters and microscope settings (Extended Data Fig. 2e,f). Objects are simulated as in the first test, except each dataset consists of only a single object, rather than a grid of objects. We generate and compare objects at um/px resolutions of 0.04, 0.06, 0.08, 0.1, 0.15, 0.2, 0.25, 0.3. For each pixel resolution, 4 objects are generated, a small (thickness of 0.4 um) and short (length of 0.4 um) object, a small and long (minimum of 49 pixels or 10 um) object, a thick (1 um) and short object, and a thick and long object, each dataset being replicated with increasing background noise values (Extended Data Fig. 2g). We calculate F1 score and IoU as before, and average the metrics across all resolutions and object sizes to generate the final quantifications (Extended Data Fig. 2e,f). As expected, a simple Otsu threshold performs best for single objects with a constant intensity against low background noise, but quickly decays, whereas Nelly performs steadily well across all noise values. MitoGraph performs consistently well, though not as well as Nellie, across all noises, except the highest in which it could not detect the object of interest. Nellie's F1 score was either better or comparable to other algorithms across all noise levels.

The final segmentation comparison tests each algorithm's ability to correctly detect and segment two adjacent objects, considering many datasets often have numerous objects of interest within closely confined regions (Extended Data Fig. 2h,i). Objects are simulated as in the second test, except no curvature is used. We again generate and compare objects at um/px resolutions of 0.04, 0.06, 0.08, 0.1, 0.15, 0.2, 0.25, 0.3, and for the 4 object dimensions as described in the second test. We additionally add in a second line during the initial skeleton population phase, separated in the X dimension

by a separation distance of 2, 2.25, and 2.5 times the thickness (Extended Data Fig. 2j). We calculate F1 score and IoU as the second test (Extended Data Fig. 2h,i). IoU and F1 performance was roughly the same for all algorithms as the second test case, with Nellie again performing steadily well throughout, though Mitograph's F1 score is slightly higher at medium-high noise levels, but drops again at the highest noise level.

Supplementary Note 5: The Swin UNETR Deep Learning Model Architecture for Organelle Segmentation in Fluorescence Microscopy Images

Here, we provide a comprehensive description of our custom-trained **Swin U-Ne**t **Tr**ansformer (Swin UNETR) models used for organelle segmentation in fluorescence microscopy images. Originally created for medical image segmentation tasks, the model leverages the Swin Transformer to capture long-range dependencies and the U-Net architecture for precise localization, making it well-suited for both 2D and 3D fluorescence microscopy image segmentation tasks[4]. The primary goal of this section is to evaluate the generalization capabilities of deep learning based organelle segmentation models when trained on limited and specific datasets, such as a single organelle type, or a single microscopy experimental setup.

We utilized the public dataset from the Allen Institute for Cell Science (AICS), from their earlier publication on label-free prediction of three-dimensional fluorescence images, where all the images we included in our training were taken from the same microscope with the same imaging settings[5]. This dataset comprises a collection of high-quality fluorescence microscopy images, each focusing on a specific cellular structure or organelle. For our experiments, we trained models on individual organelle datasets, a mitochondria-only dataset and a desmosomes-only dataset, to generate organelle specific models, as well as a combined dataset including multiple structures such as actin, actomyosin, desmosomes, endoplasmic reticulum, Golgi apparatus, mitochondria, nucleoli, tight junctions, and tubulin, to generate a microscope specific, but structure

agnostic model. Each organelle-specific dataset contains 80 images, providing a consistent set for training and validation.

Due to limited approaches for generating 3D manual segmentations at scale, and limited publicly available fluorescence-matched 3D segmentation datasets, we employed Nellie to generate ground truth segmentation masks for training (Supplementary Figure 4). While Nellie provides reliable organelle segmentation results, using its outputs as ground truth inherently limits the deep learning models to attempt to replicate Nellie's segmentation capabilities. However, as the objective of this study is specifically to evaluate the generalization capabilities of these models, rather than compare their outputs directly to Nellie, we deemed this limitation to be acceptable.

The Swin UNETR model combines the strengths of the Swin Transformer architecture with the U-Net segmentation framework, aiming to capture both global context and fine-grained details essential for accurate segmentation. Our model specifically processes 3D input patches (overlapping chunks of the original full data) of size (32, 128, 128) voxels, corresponding to the Z, Y, and X axes, and accepts single-channel grayscale images, outputting single-channel binary segmentation masks. A base feature size of 48 was selected to balance model complexity and computational efficiency.

Training was conducted separately for ten models: nine trained exclusively on individual structures–mitochondria, desmosomes, actin, actomyosin, endoplasmic reticulum, Golgi apparatus, nucleoli, tight junctions, and tubulin–two of which we showcase within this

paper, and a “combo” model trained on the combined dataset of multiple organelles (Supplementary Figure 3a). Each model was trained for 100 epochs with a batch size of 1, due to memory constraints inherent in processing 3D data, and validation scores were calculated every 5 epochs (Supplementary Figure 3b). We used a composite loss function combining Dice Loss and Cross-Entropy Loss to address class imbalance and encourage accurate voxel-wise classification.

Data augmentation techniques are crucial in attempting to enhance the models’ robustness and generalizability, given the limited size of each organelle dataset and confinement to a single microscopy setup. Augmentations included random flipping along spatial axes, random rotations, intensity normalization, padding, and cropping to standardize input dimensions.

Upon evaluation, all three models demonstrated strong performance on test data within the AICS dataset, accurately segmenting the structures they were trained on, as compared to Nellie’s “ground truth” outputs (Supplementary Figure 4a). However, when applied to fluorescence microscopy images from different sources, microscopes, or resolutions not represented in the training data, including 2D images with padding, the models exhibited significant performance degradation and segmentation artifacts were prominent (Supplementary Figure 4b,c). Additionally, even within the AICS dataset, the mitochondria-trained model appeared to oversegment non-tubular structures such as desmosomes, and similarly, the desmosome-trained model appeared to undersegment non-spherical structures, such as mitochondria and golgi. The combo model showed

some improvement across multiple structures within the combined dataset but still seemed to oversegment spherical structures (possibly due to a class imbalance issue), and struggled with data outside the AICS's microscopy setup.

These results highlight a critical limitation of deep learning models in the context of organelle segmentation: their generalization capabilities are heavily dependent on the quantity, diversity, range, and balance of the training data. When trained on datasets lacking sufficient variability in imaging conditions, organelle types, and resolutions, the models become specialized tools that perform well only within their narrow training domain.

In contrast, Nellie, being a traditional image processing algorithm, does not rely on learning from data to generalize. It applies consistent computational methods to enhance and segment structures across various datasets, imaging modalities, and resolutions. Our experiments quantitatively demonstrated that Nellie maintained robust segmentation performance when applied to simulation datasets of structures with various shapes and sizes, and qualitatively when applied to images from different microscopes, resolutions, cell types, and organelles. This consistency underscores the current advantage of traditional algorithms in scenarios where acquiring diverse and extensive training data is impractical.

The key takeaway from this study is the trade-off between specialized deep learning models and generalist algorithms like Nellie. While deep learning models have the

potential to learn complex features and achieve high accuracy within their training domain, their effectiveness diminishes when faced with data that falls outside the scope of their training. This limitation poses a significant challenge in fields like fluorescence microscopy, where imaging conditions can vary widely, and obtaining large, diverse annotated datasets is often difficult. Nellie, on the other hand, provides reliable segmentation without the need for retraining or fine-tuning, making it a valuable tool for researchers working with varied and heterogeneous datasets, especially in 3D.

Importantly, Nellie may serve as an automated way of generating ground truth segmentation masks for training, either from its raw segmentation outputs as we do here, or from a manually reinforced version of its outputs. This will hopefully lead to a deep learning model that eventually outperforms Nellie in generalizability and segmentation accuracy.

Swin UNETR Model Parameters and Training Details

In this section, we provide a detailed description of the model parameters and training configurations used in our experiments, as implemented in our code. This detailed exposition aims to facilitate replication of our work and provide insights into the parameter choices made for the Swin UNETR model in the context of organelle segmentation in fluorescence microscopy images.

The Swin UNETR model was implemented using the SwinUNETR class from the MONAI library[6]. This model architecture effectively combines Swin Transformer blocks

with a U-Net-like decoder, allowing it to capture both local and global contextual information, which is useful for accurate segmentation tasks in 3D images.

The model expects input data of any size, from 2D up to 5D data, so long as the ZYX axes are the last three dimensions. The code provided automatically selects the first slice of the non-ZYX dimensions.

The image is subsampled to chunks of dimension 32x128x128. This specific size was chosen based on the dimensions of the organelle structures in our fluorescence microscopy images and to ensure compatibility with the Swin Transformer architecture, which requires input dimensions to be divisible by the patch size and window size.

Since the fluorescence microscopy images are grayscale (single-channel), representing the intensity of fluorescence from the organelle-specific dyes, the input channel number is set to 1. The model outputs a single-channel probability map, indicating the likelihood of each voxel belonging to the organelle of interest, suitable for binary segmentation tasks, thus the output channel number is set to 1 as well. The base number of feature maps in the model is set to 48. This parameter controls the width of the network and was selected to balance between sufficient model capacity to capture complex features and computational efficiency to manage GPU memory constraints.

Other notable parameters of the SwinUNETR model were left at their default values, which are optimized for general medical image segmentation tasks. The parameters that were not explicitly modified include:

- Depths (depths): (2, 2, 2, 2)
    - Specifies the number of Swin Transformer layers at each stage of the encoder. Each number corresponds to a stage in the hierarchical architecture.
- Number of Heads (num_heads): (3, 6, 12, 24)
    - Defines the number of attention heads in the multi-head self-attention mechanism for each stage, corresponding to the depths.
- Patch Size (patch_size): (2, 4, 4)
    - Determines the size of the patches into which the input images are divided before being processed by the Swin Transformer blocks.
- Window Size (window_size): (7, 7, 7)
    - Sets the size of the local windows used in the self-attention computations within the Swin Transformer layers.

Although some of these parameters were considered for adjustment to potentially improve performance and reduce computational load, we retained the default settings after preliminary experiments indicated that they provided a good balance between accuracy and efficiency for our datasets.

We employed the DiceCELoss loss function from the MONAI library, which combines Dice Loss and Cross-Entropy Loss. Dice Loss measures the overlap between the predicted segmentation and the ground truth, which is particularly useful for addressing class imbalance in segmentation tasks. Cross-Entropy Loss evaluates the voxel-wise classification accuracy, penalizing incorrect predictions more heavily.

The combination of these two loss functions helps the model focus on both the overall shape and the precise boundaries of the organelles. The sigmoid=True parameter ensures that a sigmoid activation function is applied to the model's outputs before computing the loss, converting logits to probabilities suitable for binary classification.

We used AdamW optimizer from the PyTorch library for training, which is a variant of the Adam optimizer that includes weight decay regularization. A relatively low learning rate of 1e-4 was chosen to allow the model to converge steadily without overshooting minima. Due to the high memory requirements of processing 3D volumetric data and the complex architecture of the Swin UNETR model, we used a batch size of 1 to fit the data and model into the available GPU memory. Each model was trained for 100 epochs to ensure sufficient training iterations for the model to learn from the data. The model was evaluated on the validation set every 5 epochs to monitor its performance and to detect potential overfitting early, but as the validation process takes a substantial amount of time, we decided against a shorter validation period.

Data augmentation was performed using a series of transformations from the MONAI library to enhance the model's ability to generalize from limited data:

- Ensure Channel First (EnsureChannelFirstd)
    - Ensured that the input data has the channel dimension as the first dimension, as required by PyTorch models.
- Intensity Scaling (ScaleIntensityd)
    - Scaled the intensity values of the images to the range [0, 1], normalizing the data and helping the model to converge during training.
- Padding (SpatialPadd)
    - Applied symmetric padding to the images and labels to reach the desired spatial size (32, 128, 128). Padding was necessary because the images might not have uniform sizes and the Swin Transformer architecture requires inputs of specific dimensions.
- Random Spatial Cropping (RandSpatialCropd)
    - Extracted random crops of size (32, 128, 128) from the padded images and labels. This step helps the model learn from different regions of the images, increasing the diversity of the training data.
- Random Flipping (RandFlipd)
    - Randomly flipped the images and labels along each spatial axis (Z, Y, X) with a probability of 0.5. Flipping helps the model become invariant to the orientation of the organelles.
- Random Rotation (RandRotate90d)

    - Applied random rotations of the images and labels by 90 degrees up to three times (max_k=3) with a probability of 0.5. Rotations further enhance the model’s ability to recognize organelles in different orientations.
- Conversion to Tensors (ToTensord)
    - Converted the numpy arrays into PyTorch tensors, which are required for model input.

For validation and inference, we used the sliding_window_inference function from MONAI to handle large images that could not be processed in a single forward pass due to memory constraints

- ROI Size (roi_size): (32, 128, 128)
    - Matches the input size expected by the model.
- Sliding Window Batch Size (sw_batch_size): 1
    - Processes one window at a time during inference.
- Overlap (overlap): 0.25
    - Specifies the amount of overlap between adjacent windows to ensure seamless predictions across the entire volume.

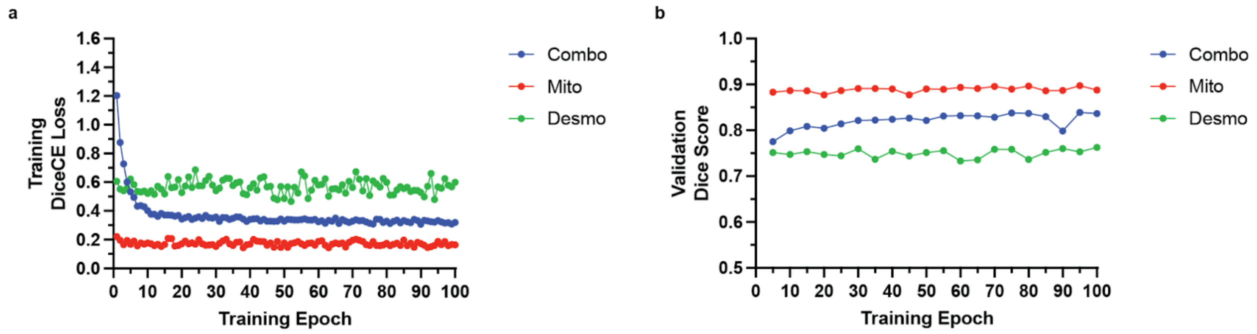

Supplementary Data Fig. 3: Training and validation metrics for Swin UNETR segmentation models
a, DiceCE loss values for training datasets of Swin UNETR models trained with datasets containing actin, actomyosin, desmosomes, endoplasmic reticulum, Golgi apparatus, mitochondria, nucleoli, tight junctions, and tubulin (blue), mitochondria alone (red) or desmosomes alone (green) at each epoch. **b**, Dice scores for training-excluded validation datasets of Swin UNETR models trained on the same respective organelle as in **a**.

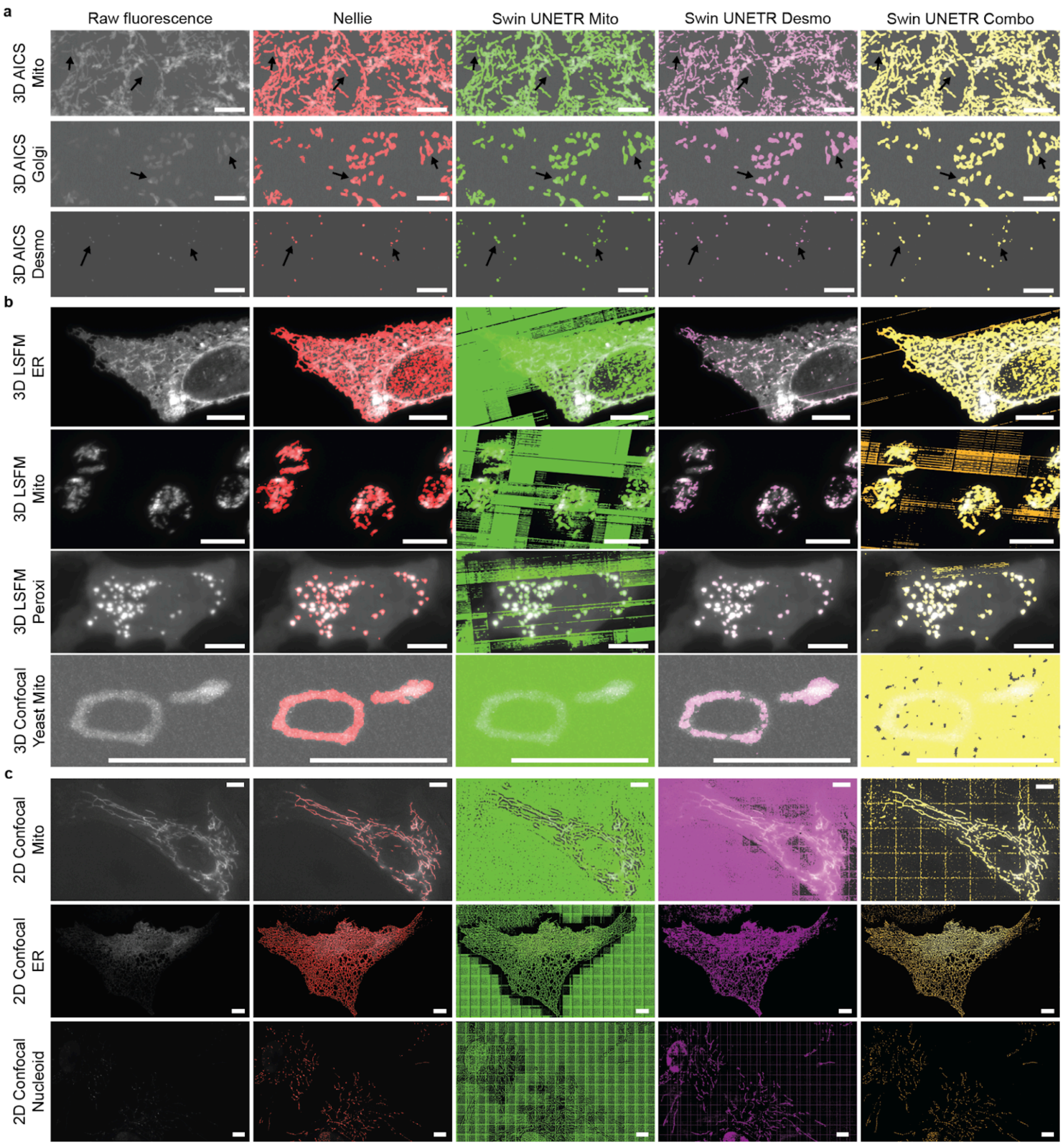

Supplementary Data Fig. 4: Visualizations of segmentations from Nellie and custom trained Swin UNETR models

Segmentation outputs from Nellie (red) and Swin UNETR models pre-trained on only mitochondria (green), only desmosomes (purple) and an aggregated dataset of actin, actomyosin, desmosomes, endoplasmic reticulum, Golgi apparatus, mitochondria, nucleoli, tight junctions, and tubulin (orange), with the raw fluorescence data shown in the left-most column. **a**, Output segmentations for the 3D dataset from the Allen Institute for Cell Science (AICS) for mitochondria, golgi, and desmosomes. Black arrows denote regions of notably different segmentation results between methods and models. **b**, Output segmentations for 3D datasets outside of the AICS dataset, including lightsheet fluorescence microscopy (LSFM) data of endoplasmic reticulum (ER), mitochondria, and peroxisomes, and confocal microscopy data of yeast cell mitochondria. **c**, Output segmentations for 2D datasets outside of the AICS dataset, including confocal microscopy datasets from mitochondria, ER, and nucleoids. Scale bars are 5 um in length.

Supplementary Note 6: Nellie's extended segmentation pipeline

The segmentation process for organelles is a simple connected-components labeling scheme. Subsequently, Nellie incorporates a binary hole-filling algorithm exclusively in the analysis of 3D images. This decision is informed by distinct considerations regarding the structural characteristics and noise levels inherent to 2D versus 3D datasets.

One of the primary reasons for omitting the hole-filling algorithm in 2D segmentation is the inherently lower level of structural noise within these datasets. The simplified nature of 2D imaging, capturing a single plane of focus, generally results in clearer delineation of organelles. This clarity reduces the likelihood of generating the types of internal inconsistencies within segmented organelles that the hole-filling algorithm is designed to address.

The decision to apply the hole-filling algorithm selectively also stems from the geometric considerations of organelles across dimensions. In 2D images, closed circular organelles are common, and applying hole-filling indiscriminately would lead to the undesired effect of filling these circles, potentially obscuring meaningful biological cavities. While the algorithm aids in creating a more consistent representation of such organelles by addressing internal voids, it operates under the assumption that spaces within the segmented organelles are artifacts rather than biologically relevant features. A notable caveat in the application of the hole-filling algorithm within 3D segmentation concerns the potential for erroneous interpretation of certain organelle geometries. Specifically, if an organelle inherently forms a spherical shape with a central void

(resembling a ball with an internal hole), the algorithm may inappropriately fill this void. This limitation underscores a challenge in distinguishing between genuine biological internalized cavities and segmentation artifacts, particularly in the context of 3D structural complexity. If spherical shapes with central voids are important for the user's analysis, we suggest replacing this binary hole filling with a binary closing operation below the scale of the object of interest.

Following thresholding, a binary opening operation is conducted to smooth out noisy or irregular boundaries of the organelles and enhance the accuracy of the subsequent segmentation by removing weak, single-voxel connections. We then use an octree-based (or quadtree in 2D) skeletonization to reduce the semantic segmentation masks to single-voxel wide representations[7,8].

Supplementary Note 7: Nellie's mocap marker generation pipeline via multi-scale and adaptive local maxima detection

To generate mocap markers, Nellie first performs a computationally efficient distance transformation over our semantic segmentation mask[9] (Supplementary Data Fig. 5, Supplementary Note 8). The distance-transformed image undergoes filtering using a Laplacian of Gaussian (LoG) filter, incorporating a set of sigma values identical to those utilized in the preprocessing pipeline. Let $I(x, y, z)$ be the input image and $L_{\sigma}(x, y, z)$ be the LoG filtered image at scale σ:

$$L_{\sigma}(x, y, z) = -\sigma^{2} \cdot \Delta\left[G_{\sigma}(x, y, z) * I(x, y, z)\right]$$

where G_{σ} is the 3D Gaussian kernel with standard deviation σ, $*$ denotes convolution, and Δ is the Laplacian operator.

The LoG filter, by design, enhances regions of rapid intensity change, which are indicative of edges and, by extension, potential peak points within the organelles. The application of this filter across multiple scales allows for the detection of peaks that correspond to features of varying sizes, ensuring comprehensive coverage of the intracellular structural landscape (Supplementary Data Fig. 6). Let R(x,y,z) be the multi-scale LoG response.

$$R(x, y, z) = max\left[L_{\sigma}(x, y, z)\right]$$

for all σ in S

where S is the set of scale values used.

Following LoG filtering, a maximum filter is applied to the resultant multi-scale filtered images. This process involves comparing each voxel to its neighbors within a defined footprint, retaining only those voxels that represent the highest value. By conducting this operation across the stacked LoG filtered images, voxels that retain their maximal value in the corresponding volume slice of the LoG filtered image are classified as local maxima for that specific scale. Let P(x,y,z) be the binary peak map.

$$P(x, y, z) = 1 \text{ if } R(x, y, z) = max[R(x', y', z')]$$

$$\text{for all } (x', y', z') \text{ in } N(x, y, z)$$

$$P(x, y, z) = 0 \text{ otherwise}$$

where $N(x, y, z)$ is the neighborhood of voxel (x,y,z).

The identified local maxima across all scales are then compiled into a single stack of images. This stack is subsequently flattened, consolidating the peak points into a singular image that serves as the basis for motion capture marker identification. This flattened image, rich in peak coordinates, effectively highlights the most prominent features within the organelles, earmarking them for feature extraction and downstream temporal linkage.

Given the potential for closely situated peaks to complicate (due to redundancy) rather than enhance subsequent analyses, an additional refinement step is employed. This step involves assessing the intensity of each peak within the original image and applying a proximity-based selection criterion. Peaks deemed too close to one another

are subjected to a comparison of their respective intensities, with preference given to the peak of higher intensity. Let Q be the set of refined peak coordinates.

$$Q = \left\{(x, y, z)\ in\ P :\ I(x, y, z) = max[I(x', y', z')]\ for\ all\ (x', y', z')\ in\ B_d(x, y, z)\right\}$$

where $B_d(x, y, z)$ is the set of peak coordinates within distance d of (x,y,z).

This is facilitated by the construction of a k-d tree from the sorted peak coordinates, enabling efficient nearest-neighbor searches to identify and eliminate proximal peaks that fall within a predefined minimum distance threshold.

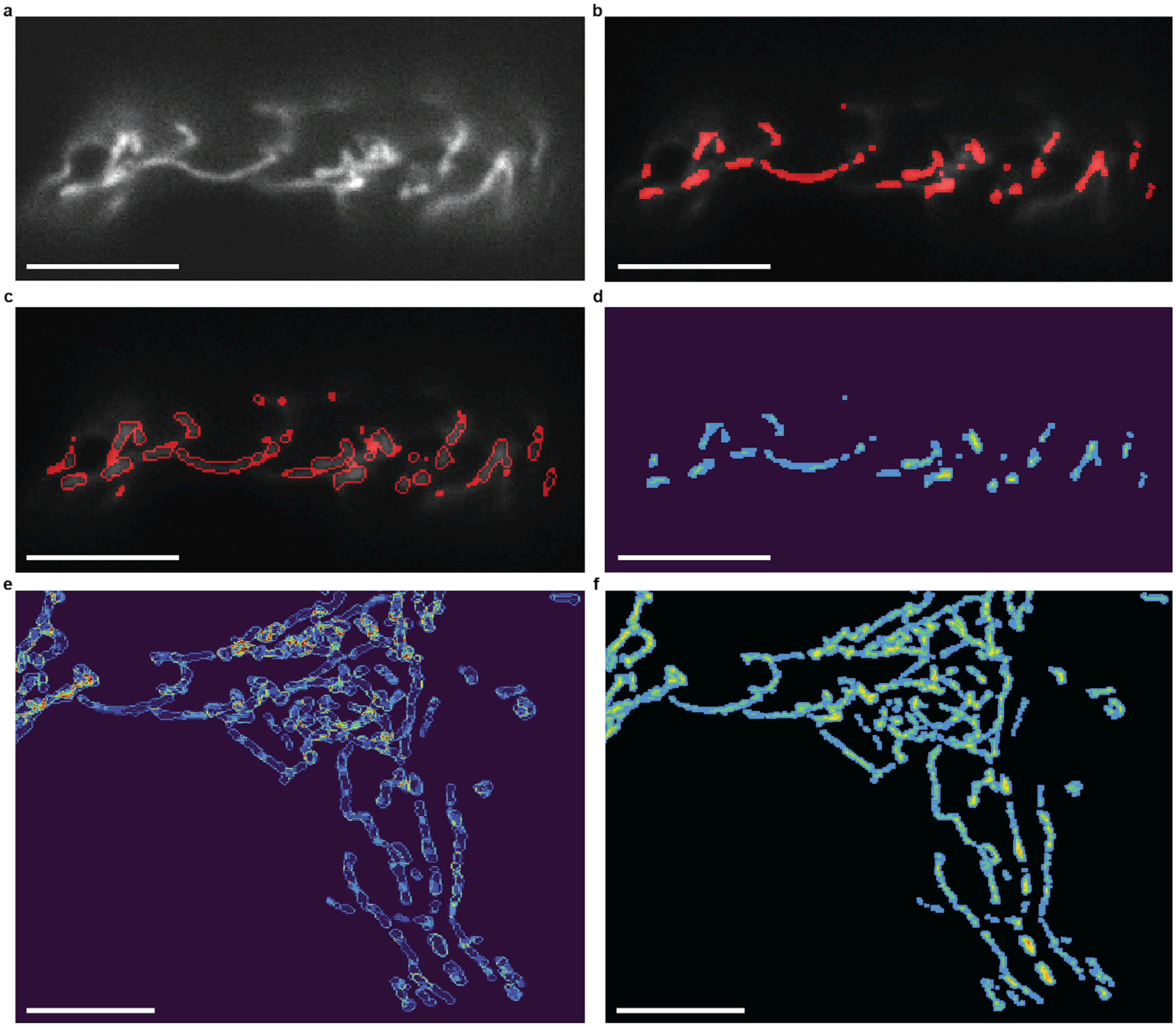

Supplementary Data Fig. 5: Computationally efficient distance transformation using segmentation border coordinate k-d trees

a, A 2D XY orthogonal slice of a 3D fluorescently mitochondrial-labeled cell from a single object lightsheet microscope (SOLS). **b**, The same 2D slice as in (**a)** of the instance segmentation of the 3D dataset. **c**, The border mask of the instance segmentation of (**b**) superimposed on (**a**), obtained via a one-voxel binary dilation of the instance segmentation mask, followed by subtraction of the instance segmentation mask. **d**, The same 2D slice as in (**a**) of the distance transformation image acquired by calculating the nearest neighbor distance of the voxel coordinates in (**b**) to the voxel coordinates in (**c**). **e**, The mean intensity projection of the 3D border mask of the instance segmentation. **f**, The max intensity projection of the 3D distance transformation image of the instance segmentation. Scale bars are 10 um in length.

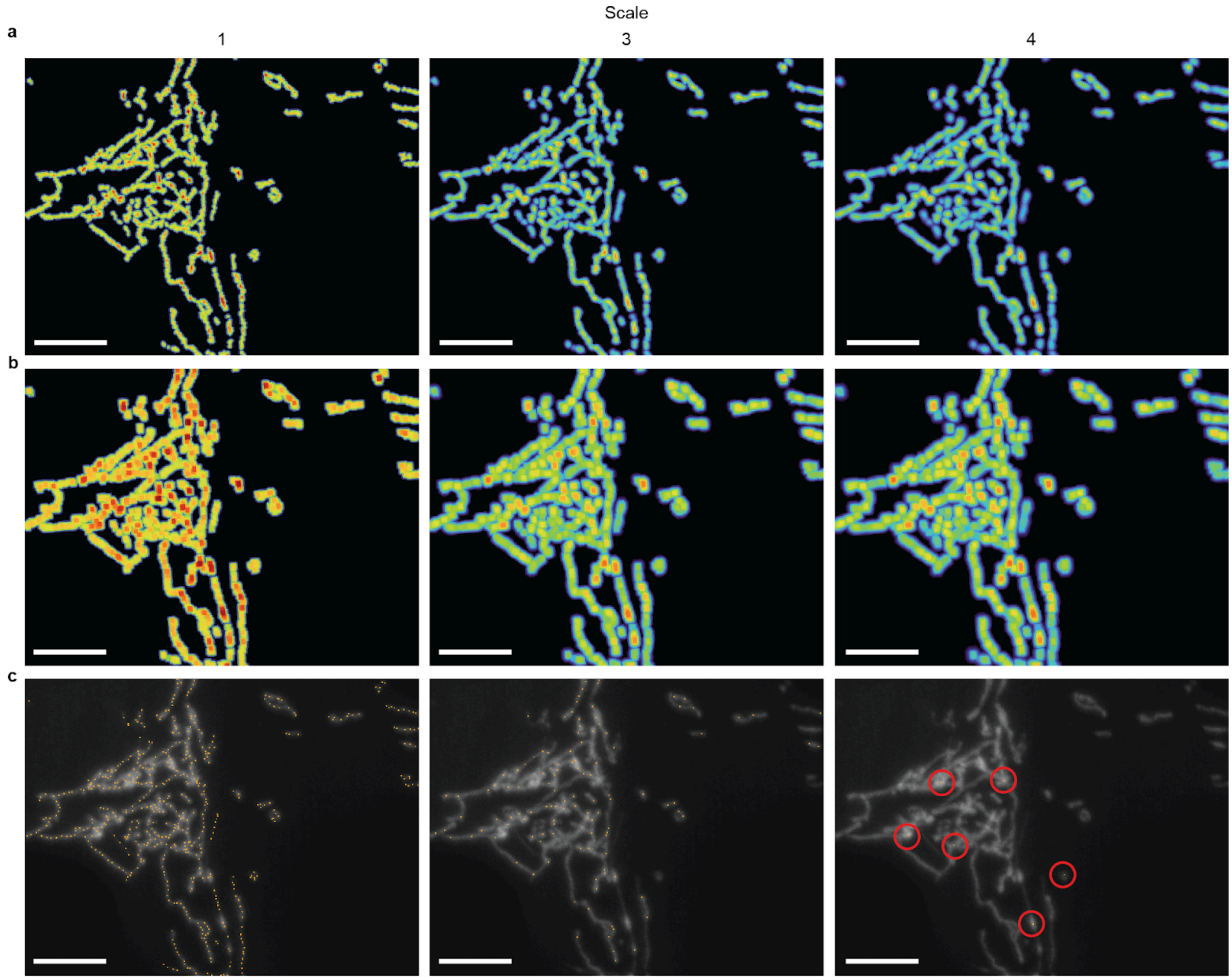

Supplementary Data Fig. 6: Multi-scale local maxima peak detection for mocap marking

a, A 3D fluorescently mitochondrial-labeled cell's post-processing volume is run through a Laplacian of Gaussian filter at different scales. Shown here is a filter sigma of 0.862, 1.67, and 2.07, (left, middle, right) corresponding to scales 1, 3, and 4, of 5 total scales. **b**, A maximum filter is run across the 4D stack of Laplacian of Gaussian filtered images (**a**). Shown here are again images corresponding to scales 1, 3, and 4, of 5 total scales. **c**, The raw fluorescence intensity image overlaid with single orange voxels corresponding to locations where the intensity value of the Laplacian of Gaussian image (**a**) is equal to the intensity value of the maximum filtered image (**b**) at different scales, again shown at scales 1, 3, and 4, of 5 total scales. Red circles are to indicate the few voxels at scale 4 where voxel intensity values match, indicating fewer (though still important) regions of larger scale structures. Scale bars are 10 um in length.

Supplementary Note 8: Efficient distance transformation using k-dimensional trees

In this section, we introduce, to our knowledge, the first application of a k-dimensional (k-d) tree for a computationally efficient distance transformation of a binary mask. The process begins with the generation of a border mask, which delineates the periphery of the organelles within the binary mask. This is accomplished by subtracting the original binary mask from its one-voxel binary dilation. Binary dilation incrementally expands the boundaries of the organelle. Let M be the original binary mask and D be the dilated mask.

$$D = M \bigoplus S$$

where ⊕ denotes morphological dilation and S is the structuring element. Let B be the border mask.

$$B = D - M$$

where $-$ denotes set difference (subtraction).

The subtraction operation thus isolates a thin border that precisely maps the outermost edge of each organelle, effectively creating a mask that represents the interface between the organelle and its surrounding environment:

Following the creation of the border mask, the next step involves constructing a k-d tree from the coordinates of the border pixels. The k-d tree is a space-partitioning data structure optimized for organizing points in a k-d space, facilitating efficient queries for nearest neighbors. In the context of our method, the k-d tree serves as a computational framework that allows for rapid identification of the closest border pixel for any given

voxel within the instance segmentation mask of organelles. Let T be the k-d tree and C_B be the set of coordinates of border pixels.

$$T = KDTree\left(C_B\right)$$

where KDTree() is the function to construct a k-d tree.

With the k-d tree in place, the final step is to perform a distance transformation on the binary mask. This is achieved by querying the k-d tree with the coordinates of all voxels in the instance mask. For each voxel, the k-d tree identifies the nearest border pixel and calculates the Euclidean distance between these two points. This process is repeated for every voxel in the mask, resulting in a distance-transformed image where the value of each voxel reflects its minimum distance to the organelle's boundary. Let DT be the distance-transformed image and C_M be the set of coordinates of mask pixels.

For each point p in C_M:

nearest_border = T.query(p, k=1)

DT(p) = EuclideanDistance(p, nearest_border)

where T.query() finds the nearest neighbor in the k-d tree, and EuclideanDistance() calculates the Euclidean distance between two points. For points p1(x1, y1, z1) and p2(x2, y2, z2):

$$\text{EuclideanDistance(p1, p2)} = \sqrt{\left(x_2 - x_1\right)^2 + \left(y_2 - y_1\right)^2 + \left(z_2 - z_1\right)^2}$$

By employing a k-d tree for the nearest-neighbor search, our approach minimizes computational overhead, enabling rapid processing of large datasets without sacrificing accuracy.

Supplementary Note 9: Tracking comparison and benchmarking against state-of-the-art organelle tracking algorithms

	Nellie (ours)	MitoTNT	Mitometer
**2D**	Yes	No	Yes
**3D**	Yes	Yes	Yes
**Metadata detection**	Yes	No	No
**Automated parameters**	Yes	Yes	Yes
**Voxel motility metrics**	Yes	No	No
**Node motility metrics**	Yes	Limited	No
**Branch motility metrics**	Yes	Limited	No
**Organelle motility metrics**	Yes	Limited	Limited
**Image-wide motility metrics**	Yes	No	No
**Object permanence**	Yes	Limited	Limited
**Track output**	Yes	Yes	Limited
**Intermediates output**	Yes	No	Limited
**Visualization**	Yes	Limited	Yes
**GUI**	Yes	No	Yes
**Language**	Python	Python	MATLAB
**Compatible OS**	Mac, Windows, Linux	Mac	Mac, Windows, Linux

Nellie, 2024 (https://github.com/aelefebv/nellie)

The subject of this paper, Nellie, is an automated segmentation, tracking, and hierarchical feature extraction pipeline for organelles in both 2D and 3D live-cell microscopy. Nellie uses its segmentation pipelines' outputs and intermediates to perform tracking. Nellie automatically detects the tracking-based metadata of the image if it is present, but allows the user to modify the metadata if needed. Nellie returns motility metrics at the voxel, node, branch, organelle, and image-wide level in .csv format. Nellie allows for maintaining object permanence (down to the sub-voxel level) using flow interpolation, meaning one can keep track of a specific voxel, node, branch, or object starting from and up to any time point. Nellie also returns all intermediates, including the distance, border, mocap marker, and frame 1 object permanence images in .ome.tif format, as well as adjacency maps for connecting adjacent frame voxels, nodes, branches, and objects in .pkl format, and mocap marker flow vector arrays in .npy format. Nellie has a Napari-based GUI, is also fully written in Python, and is compatible with all major operating systems.

MitoTNT, 2023 (https://github.com/pylattice/MitoTNT)

MitoTNT is an automated tracking, visualization, and analysis tool for 3D mitochondrial timelapse images, though it works well for other organelles as well. MitoTNT does not automatically detect the image's metadata, but does tune the tracking in response to the user-defined metadata. Though MitoTNT does not produce voxel-level outputs, it allows for the extraction of node, branch, and organelle-level motility metrics; however these outputs are limited to linear motion, such as diffusivity, using the mean squared

displacement. There is limited object permanence via a sliding-window-based graph restructuring analysis for fission and fusion detection, allowing one to link nodes and objects between frames. MitoTNT outputs a .csv listing each nodes' tracks. There are also options of converting the node tracks into fragment and segment tracks using max pooling, though we had limited success in an errorless conversion. MitoTNT does not output intermediate validation files, but does output files that allow for track and dynamics visualization via ChimeraX, a free, open-source program from UCSF (https://github.com/RBVI/ChimeraX). MitoTNT itself does not have a GUI. It is written in python, with example Jupyter notebook use cases. MitoTNT is only compatible with MacOS. To allow for an automated experience using MitoTNT, we have generated scripts for the automated conversion of timelapse images, tracking, and fission and fusion detection adapted from MitoTNT's Jupyter notebooks. The scripts and methods are accessible in the supplemental Nellie repository (https://github.com/aelefebv/nellie-supplemental).

Mitometer, 2021 (https://github.com/aelefebv/Mitometer)

Mitometer is an automated segmentation and tracking pipeline for mitochondria in both 2D and 3D live-cell microscopy, though still performs well for processing and analysis of other organelles. Mitometer uses its segmentation outputs to perform tracking. It requires the user to input temporal metadata and adjusts its tracking pipeline accordingly. Mitometer's tracking outputs are limited to connected-component center-of-mass analysis, and returns corresponding linear motion-based quantifications in a .txt file. Similar to MitoTNT, Mitometer has limited object permanence via fission and

fusion detection and subsequent object-based linkage. Mitometer has a GUI for both single-file and batch-file processing, but uses MATLAB, which is not open source, but is compatible with all major operating systems. For the sake of reproducibility in running benchmarking and comparisons, we have rewritten Mitometer's MATLAB tracking and fission and fusion algorithms in Python. The rewritten code is accessible in the supplemental Nellie repository (https://github.com/aelefebv/nellie-supplemental).

For the benchmarking and comparisons of the aforementioned tracking algorithms, we chose to compare and contrast metrics such as linear and angular velocity, track length, and fission and fusion event detection via 5 different scenarios. As generating ground truth masks and tracks of 3D + T microscopy datasets is non-trivial and subject to user bias, we instead opt to build a suite of motility simulation tools to complement the object simulation tools in the segmentation comparison section to generate ground truth organelle-like objects with known motility features. All of the simulation tools are made available in the supplemental Nellie repository (https://github.com/aelefebv/nellie-supplemental).

The first two tracking simulations test each algorithm's ability to accurately track multiple objects' motions over a number of frames (Extended Data Fig. 4a-f). To do this, we simulate a dataset consisting of 3 objects with various lengths, widths, and intensities, each moving along an orthogonal axis (i.e. X only, Y only, and Z only) at a consistent rate of 1 pixel per frame in either the direction of its long axis for 26 frames (Extended Data Fig. 4a-c), of the direction of its short axis for 28 frames (Extended Data Fig. 4d-f).

The dataset is then reversed and appended to the end of the original dataset to create a back-and-forth motion of the objects. We set a pixel resolution of 0.2 um/px and a temporal resolution of 0.5 s/frame for the original dataset, and remove temporal frames in increasing powers of 2 to generate additional datasets with temporal resolutions of 1, 2, 4, and 8 s/frame, with objects moving at a consistent speed of 0.4 um/s. We use Nellie’s features_branches.csv and im_skel_relabelled.ome.tif outputs to track individual branches over each time course, and monitor which branch we have tracked in each frame to measure how persistently each branch is tracked (Extended Data Fig. 4b,e). For MitoTNT, we attempted to use the node tracking outputs to generate fragment tracking outputs, but this process errored out in >50% of the samples. Instead, we simply use the node tracks’ outputs to calculate displacement and monitor which node we have tracked in each frame to measure how persistently each node is tracked. For Mitometer, we use the track number and its centroid at each frame to calculate the velocity, and use the appearance of each track in each frame as the measure of persistence. For both long and short axis travel, Nellie performs essentially perfectly in both linear velocity calculation, and track persistence. Mitometer also performs close to perfectly, with some variability in both velocity and track length. MitoTNT performs decently for linear velocity calculation, but seems unable to consistently keep hold of a track for longer than about 20 frames. We also note that MitoTNT was unable to produce tracks for the 8 s/frame sample in the long-axis case.

The third tracking simulation tests each algorithm's ability to quantify angular velocity of a rotating object, considering organellar movement is not limited to linear velocity alone,

but often comes about from angular movement about a pivot, such as an organellar or structural contact site. We generate a single, long object similar to how we generate an object in the first test case, but instead of linearly moving the object, we rotate the object about a single axis at a constant rate of 5 degrees per frame for 33 frames. We again set the pixel resolution to 0.2 um/px and the time interval to 0.5 s/frame, meaning the object is rotating at a constant speed of 10 deg/s about its center of mass (Extended Data Fig. 4g). We again produce additional datasets with frames at increasing powers of 2 removed, meaning the object will have rotated by 5, 10, 20, 40, and 80 degrees between each frame, for each respective dataset. We quantify both linear velocity, which should be near 0, as the object is rotating about its center of mass (though discretized to individual pixels, so it will never be exactly 0), and angular velocity, which should be a constant 10 deg/s. We use the same method as in the first test case to generate the linear velocity calculation for both Nellie and Mitometer. However, for MitoTNT, we instead use their script to convert the node tracking outputs to fragment tracks, which it was only able to do for the 1 and 2 s/frame datasets. For Nellie, we calculate angular velocity using the values in the features_nodes.csv output. Specifically, we get the mean ang_vel_mag_rel_mean, which is the mean angular velocity magnitude, relative to its pivot point, of all voxels that make up a node, and average this value over each frame. We then average each frames' average to get the final angular velocity in rad/s and convert this to deg/s. For MitoTNT, we use each node's track to calculate displacement of each node between frames, interpreted as rad/frame in this sample, and convert this to deg/s. Though this is not a perfectly accurate representation of angular velocity for MitoTNT's outputs, this was the best we

could do with their limited outputs. Mitometer has no discernable way to calculate angular velocity from its outputs. Both Nellie and Mitometer perform well at correctly finding low linear velocity values in all datasets, while MitoTNT seems to capture a higher velocity than both other algorithms at the temporal resolutions it was able to convert for fragment tracking (Extended Data Fig. 4h). Nellie performs well at a resolution of 0.5 and 1 s/frame, but performs increasingly poorly, underestimating angular velocity at lower temporal resolutions. MitoTNT performs poorly at high temporal resolution, but seems to perform increasingly better at lower temporal resolutions.

The final two tracking simulations test the algorithms' abilities to maintain object permanence by quantifying its ability to maintain a link over objects after undergoing fragmentation (fission) or merging (fusion) events (Extended Data Fig. 4j-l). We simulate 2 objects in each orthogonal plane of our image, where we extend the object from the center point of the image along either the positive or negative direction of its axis, resulting in a 6 sided star-like object. Every 3 frames, we linearly begin moving an object away from the center, along its long axis, at a rate of 1 voxel/s, again with a pixel resolution of 0.2 um/px and a temporal resolution of 0.5 s/frame. After several frames, all objects have become disconnected from the center, resulting in a total of 5 fission events over the course of 33 frames. We generate another dataset by reversing the time series, where all objects begin disconnected, but end up reconnected by the final frame, resulting in 5 fusion events over the course of 33 frames. As previously, we remove temporal frames in increasing powers of 2 to generate additional datasets with

lower temporal resolution. For Nellie, we use the organelle features' reassigned labels, and per-frame labels to track the objects of frame 1 over time. Specifically, for each frame we calculate the difference between the number of parent labels to the number of labels in that frame. If the difference in this difference value is positive between adjacent frames, this indicates a new label has appeared, whereas a negative value indicates the disappearance of a label. For MitoTNT, we run the tracking outputs through their provided code to detect fission and fusion, and sum up all rows with a fission type to count fission events, and all rows with a fusion type to count fusion events. For Mitometer, we sum up all raw detected fission events to count fission events, and all raw detected fusion events to count fusion events. Nellie perfectly detects all 5 fission and all 5 fusion events for all temporal resolutions (Extended Data Fig. 4k). MitoTNT detects at most 2 fission events and 1 fusion events, with no events detected at the lowest temporal resolutions. Mitometer picks up all 5 fission events for a few temporal resolutions, and detects no fusion events for a few temporal resolutions, detecting no events at all for the lowest temporal resolution.

Supplementary Note 10: Creation of feature-based cost matrix for inter-frame mocap marker linkage

To create the feature-based cost matrix used to link corresponding mocap markers between adjacent frames, a speed matrix is first created by calculating the displacement between all marker indices in adjacent frames (t and t+1) and dividing this by the corresponding time between frames. Let P_t and P_{t+1} be the marker positions at time t and t+1, respectively.

$$S = \frac{\left\| P_{t+1} - P_t \right\|}{t_{t+1} - t_t}$$

where ||·|| denotes the Euclidean norm.

The speeds are normalized to the maximum permissible speed, defaulting to 1 μm/sec. Let S_{max} be the maximum permissible speed:

$$S_{norm} = \frac{S}{S_{max}}$$

Any linkages exceeding the maximum distance threshold are prohibited. The speed matrix is then z-score normalized for standardization. For a matrix M:

$$Z(M) = \frac{M - \overline{M}}{std(M)}$$

Absolute value differences of the stats vectors for markers between the two frames are computed and standardized. Let V_t and V_{t+1} be the stats vectors at time t and t+1, respectively.

$$\Delta V = \left| V_{t+1} - V_t \right|$$

The resulting matrix is divided by the number of feature columns in the stats vector to provide balanced weighting in the final cost matrix. Let n_s be the number of features in the stats vector.

$$F_{norm} = \frac{Z(\Delta V)}{n_s}$$

Absolute value differences of the Hu vectors for markers between the two frames are computed and standardized. Let H_t and H_{t+1} be the Hu vectors at time t and t+1, respectively.

$$\Delta H = \left| H_{t+1} - H_t \right|$$

The Hu matrix, after normalization, is adjusted by dividing the matrix by the number of feature columns in the Hu vector, which varies depending on whether the dataset is 2D or 3D. Let n_H be the number of features in the Hu vector.

$$H_{norm} = \frac{Z(\Delta H)}{n_H}$$

The standardized distance, stats, and Hu matrices are summed to form the final cost matrix, which will be used to temporally link mocap markers:

$$C = Z\left(S_{norm}\right) + F_{norm} + H_{norm}$$

Supplementary Note 11: Temporal continuity in organelle tracking via forward and backward interpolation of semantic segmentations across frames

Nellie includes an optional, but highly requested component for tracking instance segmentations of individual organelle objects between adjacent frames. This feature allows for the extraction of motility features and changes in morphology of a specific label across multiple temporal frames. Specifically, we maintain continuity in the identification of labeled organelle voxels across frames by sequentially relabelling adjacent frames via both a forward and backward interpolation scheme from a temporal frame T of interest (Extended Data Fig. 5).

For forward relabelling from frame t to t+1, forward interpolation of motion vectors for all semantic segmentation mask voxels in frame t is performed, yielding interpolated coordinates at frame t+1. Let V_t be the set of voxel coordinates in frame t, and F_t be the flow vectors at frame t.

$$C_{t+1} = V_t + F_t\left(V_t\right)$$

where C_{t+1} are the interpolated coordinates at frame t+1.

Similarly, backward interpolation of motion vectors for all semantic segmentation mask voxels in frame t+1 is carried out, providing interpolated coordinates at frame t.

Let V_{t+1} be the set of voxel coordinates in frame t+1, and B_{t+1} be the backward flow vectors at frame t+1.

$$C_t = V_{t+1} + B_{t+1}\left(V_{t+1}\right)$$

where C_t are the interpolated coordinates at frame t.

Next, frame t voxels are matched to the closest frame t interpolated coordinates derived from frame t+1 voxels, and vice versa. Matches are only considered if they are within the maximum travel distance threshold (1 um/s). For each v in V_t and v' in V_{t+1}:

Match(v, v') if

$$\left|\left|v - C_t(v')\right|\right| < d_{max}$$

and

$$\left|\left|'v - C_{t+1}(v)\right|\right| < d_{max}$$

where d_{max} is the maximum travel distance threshold.

Unique matches are then assigned using a heap in-place priority queue based on the distance between the voxel and interpolated coordinate match. This prioritization ensures that the most accurate matches are assigned first, enhancing both computational efficiency and matching accuracy.

Let M be the set of all matches (v, v', d) where d is the distance between v and $C_{t+1}(v)$:

UniqueMatches = AssignUniqueMatches(SortByDistance(M))

where AssignUniqueMatches assigns matches using a priority queue based on distance.

Any unlabelled voxel in frame t+1 is assigned the label of its nearest relabelled voxel, provided the distance is within the maximum travel distance threshold. For each unassigned voxel u in V_{t+1}:

Label(u) = Label($argmin_v(||u - v||)$)

if $min_v(||u - v||) < d_{max}$

where v are the assigned voxels in V_{t+1}.

This relabelling process is repeated until all voxels are labeled or until the number of unlabelled voxels stabilizes between iterations:

$$\left|Unassigned_{i}\right| - \left|Unassigned_{i-1}\right| = 0$$

or

$$\left|Unassigned_{i}\right| = 0$$

where $\left|Unassigned_{i}\right|$ is the number of unassigned voxels in iteration i.

The backward relabelling process from frame t to t-1 mirrors the forward relabelling approach. First, forward and backward interpolations are conducted for frame t-1 and frame t voxels, respectively. Voxels from frame t are matched to the closest interpolated coordinates derived from frame t-1, and vice versa, within the maximum travel distance threshold. The assignment of unique matches and relabelling of unassigned voxels follows the same procedure as in forward relabelling, ensuring consistency and accuracy across temporal frames.

Supplementary Note 12: Spatial and temporal feature extraction of hierarchical organelle levels, extended

Organelle features are extracted hierarchically, with voxels representing the smallest unit of measured space, followed by nodes, which are skeleton voxels plus their surrounding radius-dependent voxels, followed by branch components, which are semantic segmentations of skeleton branches and their surrounding voxels, followed by organelles, which are semantic segmentations of connected components, followed finally by the organellar landscape, representing the entire image. Each larger component in the hierarchy also gets aggregated statistics of features from the lower levels. We have described all of the extractable features and their respective variable names in Supplementary Table 1.

At the voxel level, local flow, structure, and fluorescence intensity information is captured. At frame T, a voxel's flow is interpolated from T-1 to T, and from T to T+1 to generate its independent linear and angular velocity and acceleration vectors, of which we can break down to its magnitude and normalized orientation vectors. We also choose, for each branch, a local pivot point, defined as the voxel within a branch that has the smallest velocity vector magnitude between the two frames of interest, as well as the entire organellar landscape's center of mass, representing two different points of reference for two additional sets of linear and angular motility features. We can then extract a directionality component by calculating the voxel's motion with respect to these points of reference.

a. Linear Velocity:

$$v_{lin}(t) = \frac{r(t+1)-r(t)}{\Delta t}$$

b. Angular Velocity (3D):

$$v_{ang}(t) = \frac{r(t)\times r(t+1)}{\Delta t||r(t)\cdot r(t+1||}$$

c. Linear Acceleration:

$$a_{lin}(t) = \frac{v_{lin}(t+1)-v_{lin}(t)}{\Delta t}$$

d. Angular Acceleration:

$$a_{ang}(t) = \frac{v_{ang}(t+1)-v_{ang}(t)}{\Delta t}$$

e. Directionality:

$$D = \frac{\left|r_{ref}(t+1)\right|-\left|r_{ref}(t)\right|}{\left|r_{ref}(t+1)\right|+\left|r_{ref}(t)\right|}$$

where r_{ref} is the position relative to a reference point (local pivot or center of mass)

At the node level, we calculate neighborhood flow patterns, as well as the thickness of an organelle branch at that skeleton voxel. To extract flow patterns, we first retrieve all the flow vectors associated with the voxels of that node. To calculate the convergence of flow vectors to that node's center, we calculate the mean direction of magnitude of all the node's voxels with respect to the node center from T-1 to T. A positive number indicates voxels are flowing towards that node from T-1 to T. In contrast, divergence is calculated in the same way as convergence, but from T to T+1. A positive number indicates voxels are flowing away from that node from T to T+1. Vergere is simply the sum of divergence and convergence, where a positive number would indicate a local

bottleneck of flow, and a negative number would indicate some flow repulsion from that node. The magnitude variability of flow vectors at that node is simply the standard deviation of the node's voxels' flow vector magnitudes. The direction uniformity is the mean value of the dot product similarity matrix of the node's voxels' orientation vectors.

a. Convergence:

$$C =- \overline{\Sigma(- v_{in})d_{in}}$$

where v_{in} is the inward velocity and d_{in} is the direction to node center

b. Divergence:

$$C =- \overline{\Sigma(- v_{out})d_{out}}$$

where v_{out} is the outward velocity and d_{out} is the direction from node center

c. Vergere:

$$V = C + D$$

d. Magnitude Variability:

$$MV = std(||v||)$$

where v are the velocities of node voxels

e. Direction Uniformity:

$$DU = \overline{v_i v_j}$$

where v_i and v_j are normalized velocity vectors of node voxels

At the branch level, we calculate skeleton metrics such as length, thickness, aspect ratio, and tortuosity, as well as standard region properties such as area, axes lengths, and solidity and extent metrics. To calculate skeleton properties, we treat each network

as a graph, composed of lone tip (no adjacent), tip (one adjacent), edge (two adjacent), and junction (>2 adjacent) voxels. We modify the skeleton graph by removing all junction voxels and replacing their neighboring edge voxels with tip voxels. Each tip-to-tip segment is considered one branch instance. To efficiently traverse this graph, we construct a distance matrix between all non-zero voxels in our skeleton and link voxels that are less than 2 voxels from one another, using only the lower diagonal to avoid duplicate matches. From these linkages, we can calculate the length of a branch as the sum distance of all linkages of that branches' voxels plus the voxel-to-border distance of the tip nodes of that branch. If the branch consists of only a single lone node, we instead calculate the length of that branch to be 2 times the voxel-to-border distance of the node. From this length measurement we calculate the tortuosity of a branch, defined as its length divided by the tip-to-tip spatial distance. We calculate the thickness of a branch as its mean voxel-to-border distance times two, and its aspect ratio as its length divided by its thickness. For calculating the region's properties, we use Scikit-Image's regionprops function.

a. Branch Length:

$$L = \Sigma(d_{ij}) + d_{tip1} + d_{tip2}$$

where d_{ij} are distances between adjacent voxels and d_{tip} are tip-to-border distances

b. Tortuosity:

$$T = \frac{L}{\left\| r_{tip1} - r_{tip2} \right\|}$$

c. Thickness:

$$Th = 2\overline{d_b}$$

where d_b are voxel-to-border distances

d. Aspect Ratio:

$$AR = \frac{L}{Th}$$

At the organelle level, we simply calculate the connected component's region properties, again via Scikit-Image's regionprops function.

Finally, at the organellar landscape (image) level, we simply allow all aggregate statistics of all other hierarchies to be stored for export.

Supplementary Table 1: Table of Nellie's output features

For each level of the organellar hierarchy, we extract a multitude of features, as described in Supplementary Note 12.

Voxels:

**Feature description (units)**	**Output name**
The physical z location of that voxel (um)	z_raw
The physical y location of that voxel (um)	y_raw
The physical x location of that voxel (um)	x_raw
The fluorescence intensity image's value at that voxel (A.U.)	intensity_raw
The preprocessed image's intensity value at that voxel (A.U.)	structure_raw
Movement directionality wrt local pivot point (N/A)	directionality_rel_raw
Angular acceleration magnitude wrt local pivot point (rad/s^2)	ang_acc_mag_rel_raw
Angular acceleration magnitude, no reference point (rad/s^2)	ang_acc_mag_raw
Linear acceleration magnitude wrt local pivot point (um/s^2)	lin_acc_mag_rel_raw
Linear acceleration magnitude, no reference point (um/s^2)	lin_acc_mag_raw
Angular velocity magnitude, wrt local pivot point (rad/s)	ang_vel_mag_rel_raw
Angular velocity magnitude, no reference point (rad/s)	ang_vel_mag_raw
Linear velocity magnitude wrt local pivot point (um/s)	lin_vel_mag_rel_raw
Linear velocity magnitude, no reference point (um/s)	lin_vel_mag_raw

Nodes:

**Feature description (units)**	**Output name**
The physical z location of that node's centroid (um)	z_raw
The physical y location of that node's centroid (um)	y_raw
The physical x location of that node's centroid (um)	x_raw
Distance from the node center to the edge x2 (um)	node_thickness_raw
The sum of the convergence and divergence of vectors to the node center	vergere_raw

(um/s)	
The convergence of the flow vectors of the voxels in the node to the node center (um/s)	convergence_raw
The divergence of the flow vectors of the voxels in the node from the node center (um/s)	divergence_raw
Summary statistics of the node's voxels' metrics (see above)	voxel aggregate metrics

Branches:

**Feature description (units)**	**Output name**
The physical z location of that branch's centroid (um)	z_raw
The physical y location of that branch's centroid (um)	y_raw
The physical x location of that branch's centroid (um)	x_raw
Label corresponding to its original temporal frame 0 label, assigned during voxel reassignment, if selected (N/A)	reassigned_label_raw
The number of voxels in that branch divided by the number of voxels in that branch's convex hull (N/A)	branch_solidity_raw
The number of voxels in that branch divided by the number of voxels in that branch's bounding box (N/A)	branch_extent_raw
Minor axis length of an ellipse with the same normalized second central moments as the branch (um)	branch_axis_length_min_raw
Major axis length of an ellipse with the same normalized second central moments as the branch (um)	branch_axis_length_maj_raw
The number of voxels in the branch, scaled by the image resolution (2D: um^2 3D: um^3). In 3D this is the volume.	branch_area_raw
The branch's tips' euclidean distance divided by its length (N/A)	branch_tortuosity_raw
The length of the branch divided by its median thickness (N/A)	branch_aspect_ratio_raw
The mean thickness of the branch (um)	branch_thickness_raw
The length of the branch (um)	branch_length_raw
Summary statistics of the branch's voxels' metrics (see above)	voxel aggregate metrics
Summary statistics of the branch's nodes' metrics (see above)	node aggregate metrics

Organelles:

**Feature description (units)**	**Output name**
The physical z location of that organelle’s centroid (um)	z_raw
The physical y location of that organelle’s centroid (um)	y_raw
The physical x location of that organelle’s centroid (um)	x_raw
Label corresponding to its original temporal frame 0 label, assigned during voxel reassignment, if selected (N/A)	reassigned_label_raw
The number of voxels in that organelle divided by the number of voxels in that organelle’s convex hull (N/A)	organelle_solidity_raw
The number of voxels in that organelle divided by the number of voxels in that organelle’s bounding box (N/A)	organelle_extent_raw
Minor axis length of an ellipse with the same normalized second central moments as the organelle, scaled by the image resolution (um)	organelle_axis_length_min_raw
Major axis length of an ellipse with the same normalized second central moments as the organelle, scaled by the image resolution (um)	organelle_axis_length_maj_raw
The number of voxels in the organelle, scaled by the image resolution (2D: um^2 3D: um^3). In 3D this is the volume.	organelle_area_raw
Summary statistics of the organelle’s voxels’ metrics (see above)	voxel aggregate metrics
Summary statistics of the organelle’s nodes’ metrics (see above)	node aggregate metrics
Summary statistics of the organelle’s branches’ metrics (see above)	branch aggregate metrics

1296

1297 Image

**Feature description (units)**	**Output name**
Summary statistics of the image’s voxels’ metrics (see above)	voxel aggregate metrics
Summary statistics of the image’s nodes’ metrics (see above)	node aggregate metrics
Summary statistics of the image’s branches’ metrics (see above)	branch aggregate metrics
Summary statistics of the image’s organelles’ metrics (see above)	organelle aggregate metrics

1298

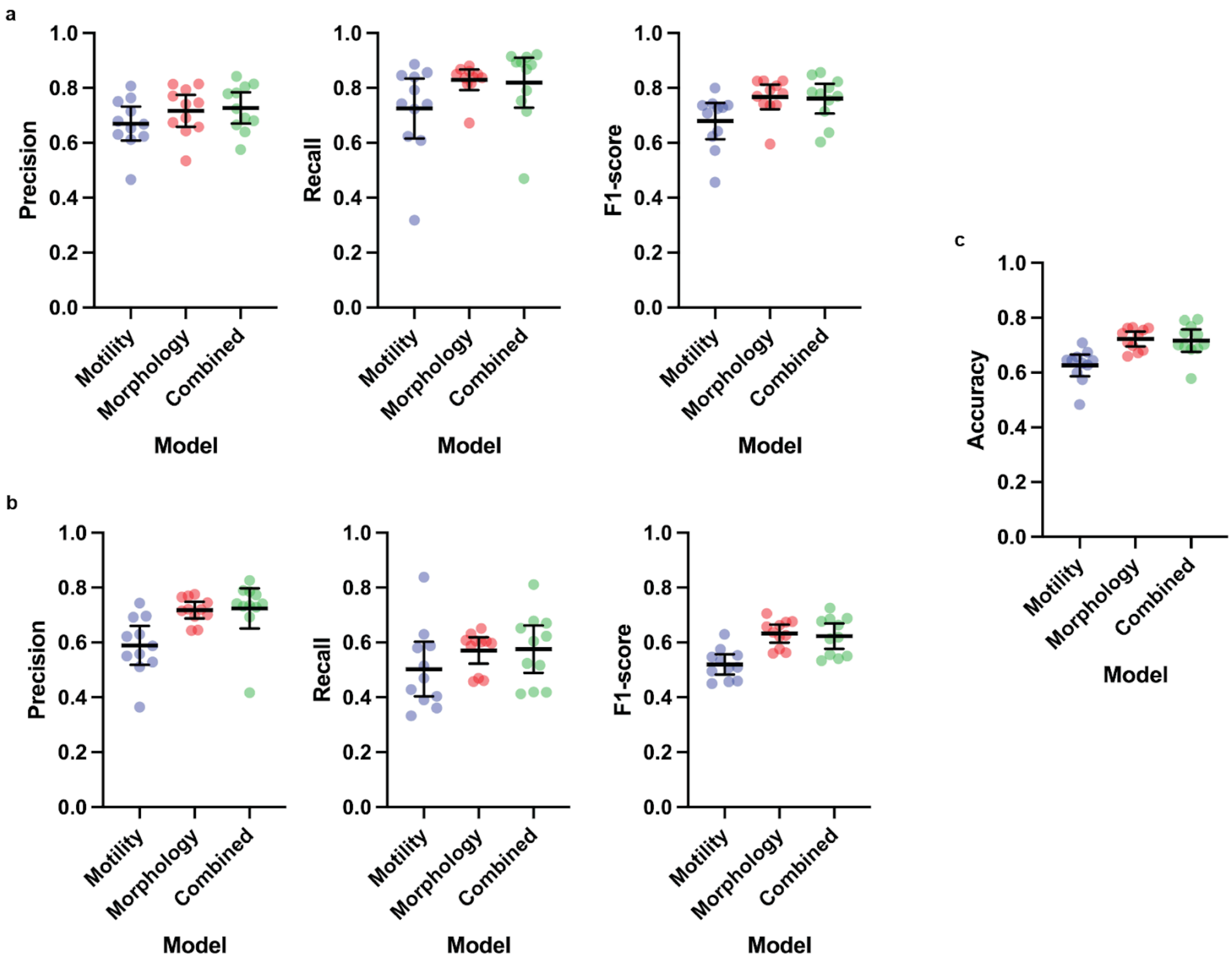

Supplementary Data Fig. 7: Additional performance metrics from organelle unmixing random forest classifiers

The precision, recall, and F1-score for Golgi (**a**) and mitochondria (**b**) classification of the motility-only, morphology-only and motility and morphology combined random forest models. **c**, The combined accuracy metric of the same three models for overall classification. N=11 leave-one-out cross validation experiments. All bars are mean +/- 95% confidence intervals.

Supplementary Note 13: Multi-mesh graph-based representation of cellular organelle networks

In the construction of the multi-mesh network from organelle segmentation masks, the methodology employs a systematic approach to transform the intricate spatial relationships of organelles into a structured graph representation. This transformation is achieved through a series of computational steps designed to map the skeletonized representations of organelles onto a graph where nodes represent skeleton voxels that carry features of their surrounding voxels, and edges that delineate the spatial connections between these nodes, capturing the underlying organizational topology of the organelle network.

The initial step involves the utilization of a k-d tree for the efficient identification of immediately neighboring nodes. This radius is chosen to ensure that all immediate neighbors in a cubic voxel grid are considered, facilitating the accurate representation of spatial adjacencies within the graph. Each node, corresponding to a skeleton voxel, is then associated with a list of its neighbors, excluding self-references, to establish the basic graph structure. For each skeleton voxel v:

$$N(v) = \frac{\{u \epsilon V : ||v-u|| \leq 1.74\}}{v}$$

where V is the set of all skeleton voxels, and ||·|| denotes Euclidean distance.

Subsequently, the algorithm identifies a starting node for the graph, preferentially selecting a node with a singular neighbor (a tip node) to anchor the construction

process. In the absence of such nodes, indicating a closed network, the first node in the branch's list is selected by default.

```
start_node = first(n for n in nodes if len(neighbors(n)) == 1)
if not start_node:
    start_node = nodes[0]
```

The construction of the multi-level mesh within the graph is then initiated by calculating the jump distances from the start node to all other nodes within the network:

$$J(v) = \min\{d : \exists \text{ path } p = \left(v_o, v_1, \dots v_d\right) \text{ where } v_0 = s, v_d = v, \text{ and } \left(v_i, v_{i+1}\right) \in E$$

$$\text{for } 0 \leq i \leq d\}$$

where s is the start node and E is the set of edges between neighboring voxels v. This calculation employs a depth-first search algorithm, incrementally increasing the jump distance as the search progresses through the network. The jump distances serve as a basis for establishing a hierarchical structure within the graph, where connections between nodes are formed based on increasing powers of two. This hierarchical connectivity facilitates efficient message passing at multiple distances, enabling the graph to capture a wide range of spatial relationships within the organelle network.

For each scale within the multi-level mesh, the algorithm generates a list of nodes that are valid at that scale, determined by their jump distances:

$$V_k = \left\{v \in V : J(v) \ \% \ 2^{k-1} = 0\right\}$$

where k is the scale level ($k \geq 1$).

This selective inclusion of nodes at each scale allows for the dynamic adjustment of the graph's hierarchy, ensuring that both local details and global organizational patterns are adequately represented. The edges within the multi-scale edge list are then generated by direct accessibility queries within the graph, establishing connections between nodes based on their proximity and mutual accessibility across the different scales of the mesh:

$$E_k = \{(u, v) : u, v \in V_k \text{ and } d(u, v) \leq k^2 + 1\}$$

where $d(u, v)$ is the shortest path distance between u and v in the original graph.

This detailed and systematic approach to constructing the multi-mesh network from organelle segmentation masks and their respective skeletons encapsulates the complex spatial organization of organelles within a scalable and interpretable graph framework. By leveraging the principles of graph theory and computational geometry, the methodology provides a robust foundation for analyzing the dynamic alterations in organelle organization, offering insights into the functional implications of spatial arrangements within cellular environments.

Supplementary Note 14: Graph autoencoder model construction for organelle multi-mesh network analysis

The graph autoencoder model is structured to transform multi-dimensional data into a comparable latent space and subsequently reconstruct the original data, aiming to retain significant biological features and spatial relationships. This allows for latent space analysis of encoded features and complex interactions within organellar networks. The graph autoencoder comprises an encoder for data compression and a decoder for data reconstruction, each built with layers specifically chosen for their effectiveness in graph-structured data processing.

The encoder initiates with a Multilayer Perceptron (MLP) to project the initial feature set into a 512-dimensional embedding. Following the MLP, a Swish (SiLU) activation function introduces non-linearity, enhancing the model's ability to capture complex patterns. Layer normalization is applied to ensure consistent training dynamics across layers by normalizing the layer outputs:

$$E(x) = \mathrm{LN}(\mathrm{SiLU}(W_e x + b_e))$$

where LN is Layer Normalization, W_e and b_e are learnable parameters.

The core of the encoder consists of 16 graph neural network (GNN) layers. These layers are designed for effective feature transformation and aggregation within the graph, utilizing mean aggregation for message passing. The independent weighting of each GNN layer allows for flexibility in learning distinct aspects of the data. Residual connections are incorporated to facilitate deeper learning without the vanishing gradient problem:

$$h_i^{(I+1)} = \mathrm{LN}(\mathrm{SiLU}\left(W_I \cdot \overline{\{h_j^I : j \in N(i)\}}\, b_i\right) + h_i^I)$$

where $N(i)$ is the set of neighbors of node i, and W_I and b_i are layer-specific parameters. For the encoder output:

$$z = h^L$$

where L is the number of GNN layers (16 in this case).

The decoder mirrors the encoder's depth but switches to graph attention network (GAT) layers:

$$a_{ij} = softmax_j\left(a^T\left[W_d h_i \,||\, W_d h_j\right]\right)$$

$$h_i^{I+1} = \mathrm{LN}(\mathrm{SiLU}\left(\Sigma_j \alpha_{ij} W_d h_j^I\right) + h_i^I)$$

where a and W_d are learnable parameters, and || denotes concatenation. For the decoder output:

$$x' = h^{L'}$$

where L' is the number of GAT layers in the decoder.

These layers leverage attention mechanisms to prioritize the most relevant features from neighboring nodes, crucial for accurately reconstructing the original feature space from the compressed latent representation. Dropout (DO) is incorporated at a rate of 20% within the GAT layers to prevent overfitting by randomly omitting subsets of features during training:

$$h_{DO} = h \cdot mask,\ mask \sim Bernoulli(p = 0.8)$$

Applied in GAT layers with 20% dropout rate.

Similar to the encoder, the decoder employs Swish activation and layer normalization post each GAT layer, concluding with a transformation back to the original feature size. This design ensures that the decoder can effectively reconstruct the original data, highlighting changes in organelle configurations.

The initial embedding is essential for transitioning from the high-dimensional raw feature space to a more comparable representation without significant information loss. The MLP efficiently achieves this transformation, while the SiLU activation function is chosen for its property of smoothing nonlinearities, improving model performance over traditional ReLU functions. The choice of GNN layers for the encoder stems from their ability to capture the dependency and interaction between intra-organellar nodes in a graph. Mean aggregation is used for its simplicity and effectiveness in summarizing variable-range neighborhood information, essential for understanding the context of each organelle node within the network. The adoption of GAT layers for the decoder is motivated by the need for a nuanced reconstruction of the original data. The attention mechanism within GAT layers allows the model to focus on the most informative parts of the input graph, crucial for detailed and accurate data reconstruction. Layer normalization and residual connection components are integral to the model for maintaining stable learning rates across training and enabling the effective training of deep networks by allowing information and gradients to flow through the network without diminishment.

The model implementation leverages PyTorch and PyTorch Geometric, facilitating efficient graph data processing and neural network operations[10,11]. Training involves minimizing the Mean Squared Error (MSE) between the original and reconstructed datasets, with the Adam optimizer chosen for its adaptive learning rate capabilities, especially suited for sparse data commonly found in graphs:

$$\text{Loss} = \text{MSE}(x, x') = \frac{1}{n}\Sigma_i \left(x_i - x'_i\right)^2$$

$$\theta_{t+1} = \frac{\theta_t - \eta \cdot m_t}{\sqrt{v_t} + \varepsilon}$$

where m_t and v_t are the first and second moments of the gradients, computed by the Adam optimizer.

For training and validation of our model, we randomly assign 70% of the graphs in each dataset for training and 30% for validation, evaluating the model on unseen data. Training uses an Adam optimizer with a 0.01 learning rate and implements early stopping to prevent overfitting. We monitor validation loss and halt training if no improvement occurs for 10 consecutive epochs, with a 0.001 minimum improvement threshold. We plot and compare training and validation loss curves to visually identify divergence indicating overfitting. We prevent data leakage by strictly separating training and validation datasets, performing normalization and feature scaling independently on each set. We use a batch size of 1 to process each graph individually, avoiding information sharing between samples. The model's performance is continuously evaluated on the validation set, with the best-performing model saved based on lowest validation loss (Supplementary Data Fig. 8). This approach to training and validation,

coupled with checks for overfitting and data leakage, ensures the integrity and reliability of our GNN Autoencoder model.

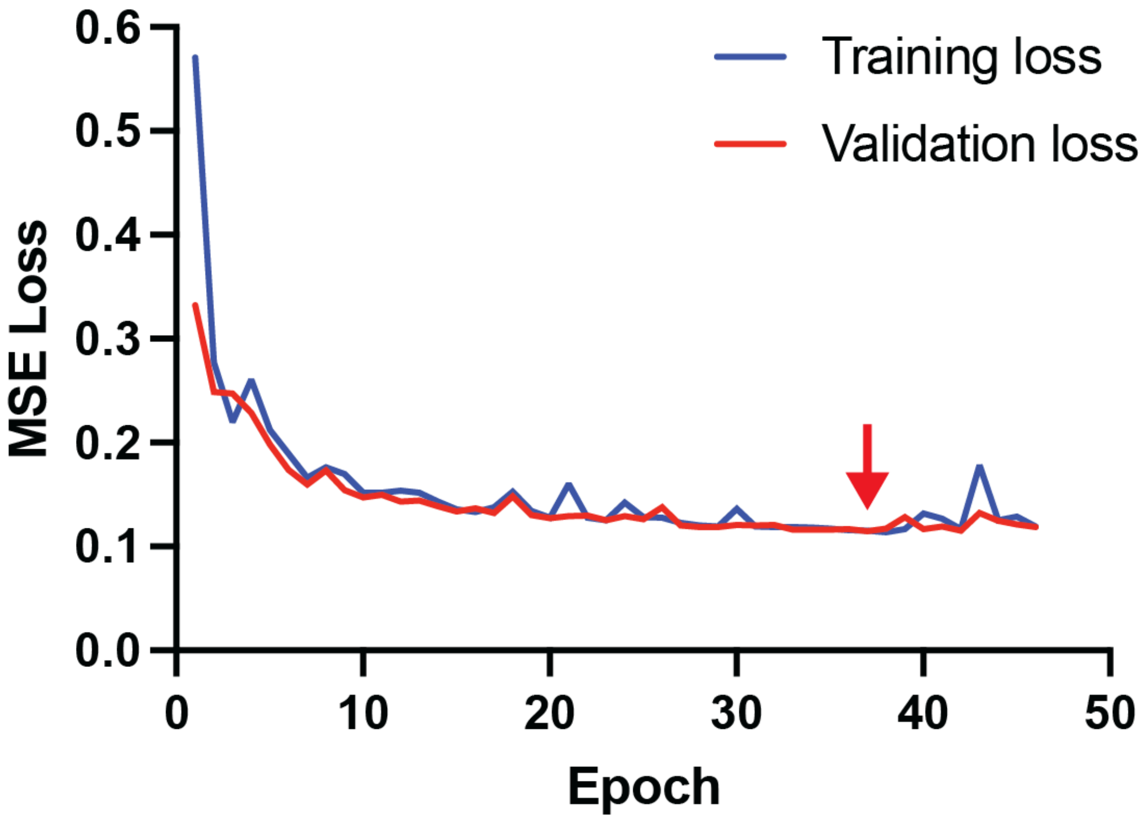

Supplementary Data Fig. 8: Loss curves for the Multi-mesh GNN

Loss curves for the multimesh GNN are shown for training (blue) and validation (red) datasets. The model was trained for a total of 47 epochs. The model was saved for the lowest validation loss epoch (37, red arrow).

References

1. Otsu, N. A Threshold Selection Method from Gray-Level Histograms. *IEEE Trans. Syst. Man Cybern.* **9**, 62–66 (1979).
2. Zack, G. W., Rogers, W. E. & Latt, S. A. Automatic measurement of sister chromatid exchange frequency. *J. Histochem. Cytochem.* **25**, 741–753 (1977).
3. Viana, M. P., Lim, S. & Rafelski, S. M. Quantifying mitochondrial content in living cells. *Methods Cell Biol.* **125**, 77–93 (2015).
4. Hatamizadeh, A. *et al.* Swin UNETR: Swin Transformers for Semantic Segmentation of Brain Tumors in MRI Images. Preprint at https://doi.org/10.48550/arXiv.2201.01266 (2022).
5. Ounkomol, C., Seshamani, S., Maleckar, M. M., Collman, F. & Johnson, G. R. Label-free prediction of three-dimensional fluorescence images from transmitted light microscopy. 289504 Preprint at https://doi.org/10.1101/289504 (2018).
6. Consortium, M. MONAI: Medical Open Network for AI. Zenodo https://doi.org/10.5281/zenodo.12542217 (2024).
7. Lee, T. C., Kashyap, R. L. & Chu, C. N. Building Skeleton Models via 3-D Medial Surface Axis Thinning Algorithms. *CVGIP Graph. Models Image Process.* **56**, 462–478 (1994).
8. Meagher, D. Geometric modeling using octree encoding. *Comput. Graph. Image Process.* **19**, 129–147 (1982).
9. Borgefors, G. Distance transformations in arbitrary dimensions. *Comput. Vis. Graph. Image Process.* **27**, 321–345 (1984).
10. Paszke, A. *et al.* PyTorch: An Imperative Style, High-Performance Deep Learning Library. Preprint at https://doi.org/10.48550/arXiv.1912.01703 (2019).
11. Fey, M. & Lenssen, J. E. Fast Graph Representation Learning with PyTorch Geometric. Preprint at https://doi.org/10.48550/arXiv.1903.02428 (2019).
