# Supplementary material for: Nellie: automated organelle segmentation, tracking and hierarchical feature extraction in 2D/3D live-cell microscopy
Source: Nat Methods. 2025 Feb 27;22(4):751–63. doi: 10.1038/s41592-025-02612-7 (PMC11978511; doi:10.1038/s41592-025-02612-7)
Supplement: Supplementary file 2 — Reporting Summary [file 41592_2025_2612_MOESM2_ESM.pdf]

nature portfolio

Corresponding author(s): Austin E. Y. T. Lefebvre

Last updated by author(s): Dec 3, 2024

Reporting Summary

Nature Portfolio wishes to improve the reproducibility of the work that we publish. This form provides structure for consistency and transparency in reporting. For further information on Nature Portfolio policies, see our Editorial Policies and the Editorial Policy Checklist.

Statistics

For all statistical analyses, confirm that the following items are present in the figure legend, table legend, main text, or Methods section.

n/a	Confirmed	
☐	☒	The exact sample size (*n*) for each experimental group/condition, given as a discrete number and unit of measurement
☐	☒	A statement on whether measurements were taken from distinct samples or whether the same sample was measured repeatedly
☐	☒	The statistical test(s) used AND whether they are one- or two-sided<br>*Only common tests should be described solely by name; describe more complex techniques in the Methods section.*
☒	☐	A description of all covariates tested
☐	☒	A description of any assumptions or corrections, such as tests of normality and adjustment for multiple comparisons
☐	☒	A full description of the statistical parameters including central tendency (e.g. means) or other basic estimates (e.g. regression coefficient) AND variation (e.g. standard deviation) or associated estimates of uncertainty (e.g. confidence intervals)
☐	☒	For null hypothesis testing, the test statistic (e.g. *F*, *t*, *r*) with confidence intervals, effect sizes, degrees of freedom and *P* value noted<br>*Give P values as exact values whenever suitable.*
☒	☐	For Bayesian analysis, information on the choice of priors and Markov chain Monte Carlo settings
☒	☐	For hierarchical and complex designs, identification of the appropriate level for tests and full reporting of outcomes
☒	☐	Estimates of effect sizes (e.g. Cohen's *d*, Pearson's *r*), indicating how they were calculated

Our web collection on statistics for biologists contains articles on many of the points above.

Software and code

Policy information about availability of computer code

Data collection

The Nellie pipeline and its Napari-based plugin is fully written in Python. The Python code and plugin are freely available online via Github at https://github.com/aelefebv/nellie. Supplemental materials for non pipeline-related code can be found via Github at https://github.com/aelefebv/nellie-supplemental. A template for creating Nellie plugins can be found via GitHub at https://github.com/aelefebv/nellie-plugin-example.

Main:
numpy==1.26.4
scipy==1.12.0
scikit-image==0.22.0
nd2==0.9.0
ome-types==0.5.2
pandas==2.2.1
matplotlib==3.8.3
napari[all]==0.4.19.post1
imagecodecs==2024.9.22
pydantic==2.9.2
pydantic-core==2.23.4

Supplementary:
alabaster==0.7.16
annotated-types==0.6.0

```
app-model==0.2.6
appdirs==1.4.4
appnope==0.1.4
asttokens==2.4.1
attrs==23.2.0
Babel==2.14.0
build==1.2.1
cachey==0.2.1
certifi==2024.2.2
charset-normalizer==3.3.2
click==8.1.7
cloudpickle==3.0.0
comm==0.2.2
contourpy==1.2.1
cycler==0.12.1
czifile==2019.7.2
dask==2024.4.1
debugpy==1.8.1
decorator==5.1.1
docstring_parser==0.16
docutils==0.20.1
exceptiongroup==1.2.0
executing==2.0.1
fastdist==1.1.6
fonttools==4.53.1
freetype-py==2.4.0
fsspec==2024.3.1
HeapDict==1.0.1
hsluv==5.0.4
idna==3.7
igraph==0.11.6
imageio==2.34.0
imagesize==1.4.1
importlib_metadata==7.1.0
in-n-out==0.2.0
ipykernel==6.29.4
ipython==8.23.0
jedi==0.19.1
Jinja2==3.1.3
joblib==1.4.2
jsonschema==4.21.1
jsonschema-specifications==2023.12.1
jupyter_client==8.6.1
jupyter_core==5.7.2
kiwisolver==1.4.5
lazy_loader==0.4
llvmlite==0.42.0
locket==1.0.0
magicgui==0.8.2
markdown-it-py==3.0.0
MarkupSafe==2.1.5
matplotlib==3.9.2
matplotlib-inline==0.1.6
mdurl==0.1.2
napari==0.4.19.post1
napari-console==0.0.9
napari-plugin-engine==0.2.0
napari-plugin-manager==0.1.0a2
napari-svg==0.1.10
nest-asyncio==1.6.0
networkx==3.3
npe2==0.7.5
numba==0.59.1
numpy==1.26.4
numpydoc==1.7.0
ome-types==0.5.1.post1
packaging==24.0
pandas==2.2.2
parso==0.8.4
partd==1.4.1
pexpect==4.9.0
pillow==10.3.0
Pint==0.23
platformdirs==4.2.0
pooch==1.8.1
prompt-toolkit==3.0.43
```

psutil==5.9.8
psygnal==0.11.0
ptyprocess==0.7.0
pure-eval==0.2.2
pyconify==0.1.6
pydantic==2.7.0
pydantic-compat==0.1.2
pydantic_core==2.18.1
Pygments==2.17.2
PyOpenGL==3.1.7
pyparsing==3.1.2
pyproject_hooks==1.0.0
PyQt5==5.15.10
PyQt5-Qt5==5.15.13
PyQt5-sip==12.13.0
python-dateutil==2.9.0.post0
pytz==2024.1
PyYAML==6.0.1
pyzmq==25.1.2
qtconsole==5.5.1
QtPy==2.4.1
referencing==0.34.0
requests==2.31.0
rich==13.7.1
rpds-py==0.18.0
scikit-image==0.23.1
scikit-learn==1.5.1
scipy==1.13.0
seaborn==0.13.2
shellingham==1.5.4
six==1.16.0
snowballstemmer==2.2.0
Sphinx==7.2.6
sphinxcontrib-applehelp==1.0.8
sphinxcontrib-devhelp==1.0.6
sphinxcontrib-htmlhelp==2.0.5
sphinxcontrib-jsmath==1.0.1
sphinxcontrib-qthelp==1.0.7
sphinxcontrib-serializinghtml==1.1.10
stack-data==0.6.3
superqt==0.6.3
tabulate==0.9.0
tensorly==0.8.1
texttable==1.7.0
threadpoolctl==3.5.0
tifffile==2024.2.12
tomli==2.0.1
tomli_w==1.0.0
toolz==0.12.1
tornado==6.4
tqdm==4.66.2
traitlets==5.14.2
typer==0.12.3
typing_extensions==4.11.0
tzdata==2024.1
urllib3==2.2.1
vispy==0.14.2
wcwidth==0.2.13
wrapt==1.16.0
xsdata==24.3.1
zipp==3.18.1

Data analysis

The Nellie pipeline and its Napari-based plugin is fully written in Python. The Python code and plugin are freely available online via Github at https://github.com/aelefebv/nellie. Supplemental materials for non pipeline-related code can be found via Github at https://github.com/aelefebv/nellie-supplemental. A template for creating Nellie plugins can be found via GitHub at https://github.com/aelefebv/nellie-plugin-example.

For manuscripts utilizing custom algorithms or software that are central to the research but not yet described in published literature, software must be made available to editors and reviewers. We strongly encourage code deposition in a community repository (e.g. GitHub). See the Nature Portfolio guidelines for submitting code & software for further information.

Data

Policy information about availability of data

All manuscripts must include a data availability statement. This statement should provide the following information, where applicable:

- Accession codes, unique identifiers, or web links for publicly available datasets
- A description of any restrictions on data availability
- For clinical datasets or third party data, please ensure that the statement adheres to our policy

The authors declare that all data supporting the findings of this study are available in the article and its supplementary information files, and raw .tif files are available upon request only, due to the large size and number of tif files present throughout the manuscript. Example images are provided within the GitHub repo for testing at https://github.com/aelefebv/nellie/tree/main/sample_data. Source data for figures are provided with this paper.

Human research participants

Policy information about studies involving human research participants and Sex and Gender in Research.

Reporting on sex and gender	No data from human research participants have been collected for this study.
Population characteristics	N/A
Recruitment	N/A
Ethics oversight	N/A

Note that full information on the approval of the study protocol must also be provided in the manuscript.

Field-specific reporting

Please select the one below that is the best fit for your research. If you are not sure, read the appropriate sections before making your selection.

☒ Life sciences ☐ Behavioural & social sciences ☐ Ecological, evolutionary & environmental sciences

For a reference copy of the document with all sections, see nature.com/documents/nr-reporting-summary-flat.pdf

Life sciences study design

All studies must disclose on these points even when the disclosure is negative.

Sample size	Sample sizes were chosen to give a reasonable visualization of Nellie's pipeline (Figs. 1-3).<br>Sample sizes were chosen to replicate a typical single-cell imaging experiment (Fig. 4).<br>Sample sizes were chosen to replicate a typical long 3D lightsheet timelapse imaging experiment (Fig. 5)<br>Sample sizes were chosen to showcase exactly 2 cell types in 3 timepoints (Fig. 6)
Data exclusions	No data were excluded from analyses.
Replication	For multi-organelle unmixing, an 11-fold leave one out cross validation was used as replicates. For multi-mesh graph experiments, the graph neural net was retrained multiple times with similar results for each. The experiments were replicated twice. For simulations, one run was performed as the methods used are deterministic (Ext. Fig. 1), and one simulation set of all conditions listed were performed for each method (Ext. Fig. 2 and Ext. Fig. 4)
Randomization	Randomization was not relevant to our studies as there were no inherently randomizable conditions.
Blinding	Blinding was not relevant to our studies as no manual or user-biased selection of datasets or analysis was applicable to the presented figures or results.

Reporting for specific materials, systems and methods

We require information from authors about some types of materials, experimental systems and methods used in many studies. Here, indicate whether each material, system or method listed is relevant to your study. If you are not sure if a list item applies to your research, read the appropriate section before selecting a response.

Materials & experimental systems

n/a	Involved in the study
☒	☐ Antibodies
☐	☒ Eukaryotic cell lines
☒	☐ Palaeontology and archaeology
☒	☐ Animals and other organisms
☒	☐ Clinical data
☒	☐ Dual use research of concern

Methods

n/a	Involved in the study
☒	☐ ChIP-seq
☒	☐ Flow cytometry
☒	☐ MRI-based neuroimaging

Eukaryotic cell lines

Policy information about cell lines and Sex and Gender in Research

Cell line source(s)	U-2 OS cell line was purchased through ATCC (#HTB96), hFB cell line was purchased through LifeLine Cell Technologies (FC-0024)
Authentication	The cell lines were not authenticated
Mycoplasma contamination	The cell lines were not tested for mycoplasma contamination
Commonly misidentified lines (See ICLAC register)	No commonly misidentified cell lines were used in this study.
